# Adaptive genomic evolution of opsins reveals that early mammals flourished in nocturnal environments

**DOI:** 10.1186/s12864-017-4417-8

**Published:** 2018-02-05

**Authors:** Rui Borges, Warren E. Johnson, Stephen J. O’Brien, Cidália Gomes, Christopher P. Heesy, Agostinho Antunes

**Affiliations:** 1 CIIMAR/CIMAR, Interdisciplinary Centre of Marine and Environmental Research, University of Porto, Terminal de Cruzeiros do Porto de Leixões, Av. General Norton de Matos, s/n, 4450–208, Porto, Portugal; 2 Department of Biology, Faculty of Sciences, University of Porto, Rua do Campo Alegre, 4169-007 Porto, Portugal; 3 Smithsonian Conservation Biology Institute, National Zoological Park, 1500 Remount Road, Front Royal, VA 22630 USA; 4 Theodosius Dobzhansky Center for Genome Bioinformatics, St. Petersburg State University, St. Petersburg, Russia 199004; 5 Guy Harvey Oceanographic Center, Halmos College of Natural Sciences and Oceanography, Nova Southeastern University, 8000, North Ocean Drive, Ft Lauderdale, 33004 Florida USA; 6 ICBAS, Institute of the Biomedical Sciences of Abel Salazar, University of Porto, Rua de Jorge Viterbo Ferreira, 228, 4050-313 Porto, Portugal; 7 Department of Anatomy, Arizona College of Osteopathic Medicine, Midwestern University, 19555 N. 59th avenue, Glendale, AZ USA

**Keywords:** Nocturnal bottleneck, Mammals, Opsins, Nocturnal lifestyle, Ultra-violet sensitive vision, Panoramic vision, Visual acuity

## Abstract

**Background:**

Based on evolutionary patterns of the vertebrate eye, Walls (1942) hypothesized that early placental mammals evolved primarily in nocturnal habitats. However, not only Eutheria, but all mammals show photic characteristics (i.e. dichromatic vision, rod-dominated retina) suggestive of a scotopic eye design.

**Results:**

Here, we used integrative comparative genomic and phylogenetic methodologies employing the photoreceptive opsin gene family in 154 mammals to test the likelihood of a nocturnal period in the emergence of all mammals. We showed that mammals possess genomic patterns concordant with a nocturnal ancestry. The loss of the *RH2*, *VA*, *PARA*, *PARIE* and *OPN4x* opsins in all mammals led us to advance a probable and most-parsimonious hypothesis of a global nocturnal bottleneck that explains the loss of these genes in the emerging lineage (> > 215.5 million years ago). In addition, ancestral character reconstruction analyses provided strong evidence that ancestral mammals possessed a nocturnal lifestyle, ultra-violet-sensitive vision, low visual acuity and low orbit convergence (i.e. panoramic vision).

**Conclusions:**

Overall, this study provides insight into the evolutionary history of the mammalian eye while discussing important ecological aspects of the photic paleo-environments ancestral mammals have occupied.

**Electronic supplementary material:**

The online version of this article (10.1186/s12864-017-4417-8) contains supplementary material, which is available to authorized users.

## Background

Walls, in 1942, first recognized that placental mammals have anatomical eye and retinal characteristics that are suitable to an ancestral nocturnal lifestyle and introduced the nocturnal bottleneck hypothesis: “*We can be sure that at an early period in a placentalian evolution, the only placentals on earth were so thoroughly nocturnal…*” [1]. Supported by very detailed comparative-anatomy studies in mammals and other vertebrates, Walls demonstrated that eutherians possess markedly spherical eyes and do not have oil droplets – retinal components which facilitate colour discrimination [2]. However, not only Eutheria, but all mammals present photic-related structures that suggest past adaptation to nocturnal environments. For example, mammals have fewer photoreception organs, and therefore rely on the eye as the sole photoreceptive organ [3, 4]. In contrast with diurnal Tetrapoda, which are mostly tetrachromatic (possess four types of cone-cells involved in colour vision), mammals are mostly dichromatic and thus less able to discriminate colours. In addition, mammals have a clear preponderance of rod cells in the retina [5, 6], which perform notably well in low-light conditions [5]. Furthermore, the mammalian eye design is in some aspects indicative of being scotopic adapted (i.e. low-light adapted) [7]. Mammals present the highest convergence of orbital disposition (facing in a similar direction) among amniotes and thus the largest binocular visual fields [8], which indicates better perception of object texture, greater discrimination of contrast, and improved light sensitivity [8, 9]. Mammals also possess higher relative corneal diameters [7], presumably improving visual sensitivity under scotopic light conditions [1], and smaller absolute axial eye length, particular when compared with birds [10]. Higher axial eye length has been associated with higher visual acuity and thus with photopic-adapted (i.e. well-lit adapted) eyes [1, 8].

At the molecular level, mammalian photoreception is mediated by opsins (Table 1), which are hepta-transmembrane proteins involved in the detection of photic stimuli [11, 12]. Compared with other vertebrates, mammals have lost two visual opsins: it was reported that *RH2* is missing in all mammals, while therians have lost the *OPN1sw2* and monotremes the *OPN1sw1* [5]; furthermore, one representative of the melanopsin subfamily (*OPN4x*) is absent in mammals [13, 14].

**Table 1:** Tetrapoda opsins and their functions. Subfamilies characterization according to [5, 12, 78] and [4]

Subfamily	Opsins	Functions
Visual opsins	*RH1* *RH2* *OPN1sw1* *OPN1sw2* *OPN1lw*	Rhodopsin mediates vision in dim-light whereas conopsins are responsible for colour vision. *OPN1lw* is sensitive to red-green or long-wavelengths, *RH2* to green or middle wavelengths and two short-wave conopsins (*OPN1sw2* and *OPN1sw1*) respond to blue-violet or violet-ultraviolet wavelengths, respectively.
Non-visual opsins (sensu stricto)	*OPN3* *TMT* *TMT2*	*TMT* (teleost-multiple-tissue) are expressed in the liver, kidney and heart as well as eye and brain. Phylogenetic analysis reveals that it clades with *OPN3*, which also exhibits a multiple patterns of tissue expression.
Pineal opsins	*PARA* *PARIE* *PIN* *VA*	Multiple opsins (*PARA*, *PARIE*, *PIN*) have been isolated from the parapineal complex. *VA* opsin is also expressed in the retina and was shown that forms a functional photopigment sensitive in the 460–480 nm range.
Photoisomerases and Neuropsins	*OPN5* *RGR* *RRH*	*RGR* and *RRH* have a probable role as all-trans retinal photoisomerases. *OPN5* shows an absorption maximum at 380 nm and is thus UV-sensitive.
Melanopsins	*OPN4x* *OPN4m*	Melanopsins are involved in circadian rhythm regulation and pupillary light reflexes.

While a nocturnal ancestry appears possible for all mammals, and not only for placentals as Walls firstly assumed [1], a proper phylogenetic approach conjugating ecological, macro-evolutionary, and molecular photic adaptations of mammals still needs to be implemented. Given the central role of opsins mediating photic-related responses, they are ideal genes to retrace the evolution of the mammalian eye. Here, we used integrative comparative genomics and evolutionary methodologies employing the opsin gene family across ~154 mammals to test the likelihood of a global nocturnal bottleneck.

## Results

### Synteny analysis of tetrapod opsins

**Fig. 1:**
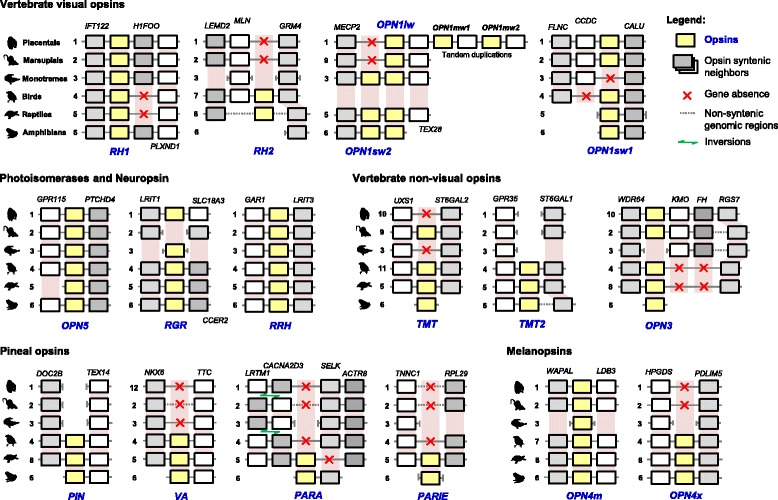
Opsin syntenic patterns in Tetrapoda. Assembled genomes were inspected for the opsin genes (yellow boxes) and their neighbouring genes (grey shaded boxes) on both sides. Segmented lines indicate genomic regions with no homologous representative between the analysed genomes. Shaded rectangles express the expected location of the analysed genes in the tetrapod genomes, pointing out the potential and confirmed losses of opsins (i.e. lack of a homologous gene in a homologous region). Missing opsin genes are identified by a red x. Species index: 1. Human (*Homo sapiens*), 2. Opossum (*Monodelphis domestica*), 3. Platypus (*Ornitorhynchus anatinus*), 4. Zebrafinch (*Taeniopygia guttata*), 5. Carolina anole (*Anolis carolinensis*), 6. Xenopus (*Xenopus laevis*), 7. Chicken (*Gallus gallus*), 8. Chinese turtle (*Pelodiscus sinensis*), 9. Tasmanian devil (*Sarcophilus harrisii*), 10. Chimpanzee (*Pan troglodytes*), 11. Flycatcher (*Ficedula albicollis*) and 12. Orangutan (*Pongo abelii*)

tblastn searches in mammalian and non-mammalian tetrapod genomes were performed to assess opsins presence and absence [15, 16]. We confirmed that amphibians, reptiles and birds have 16, 17 and 15 opsin genes, respectively, while only 10 opsin genes were identified in mammals (*RH1*, *OPN1sw1*, *OPN1sw2*, *OPNlw*, *OPN3*, *TMT*, *OPN5*, *RGR*, *RRH* and *OPN4m*). Further exploration of the absent mammalian opsins among the tetrapod genomes (Fig. 1) showed the existence of a conserved synteny in the genomic regions where *RH2*, *VA*, *PARA*, *PARIE* and *OPN4x* opsins were located, suggesting that these opsins were lost in mammals. The same could not be advanced to the *PIN* and *TMT2* opsins due to the inconsistent syntenic patterns of these genomic regions (Fig. 1). According to the syntenic analysis, mammals differ in their repertoire of opsins: monotremes were the only lineage with *OPN1sw2*, but not *OPN1sw1,* which was only present in therians; *TMT* was only present in marsupials. We also observed that *RGR* was absent in marsupials and, *OPN3* was absent in monotremes; however, due to evidence of genomic rearrangements in these regions, these absences could not be syntenic validated as lineage-specific losses (Fig. 1).

### Site- and branch-selection in mammalian opsins

**Fig. 2:**
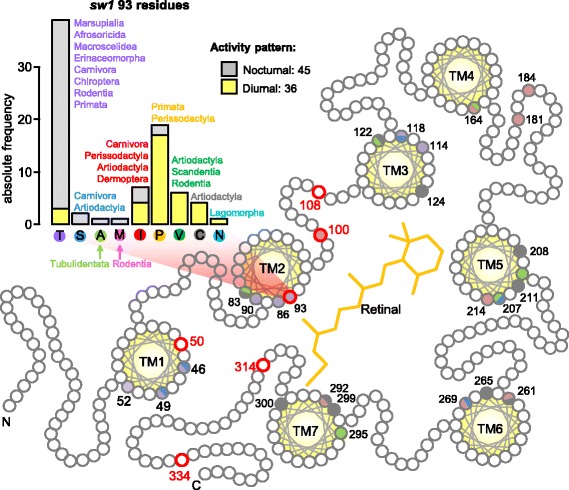
Selective signatures in the mammalian *OPN1sw1* opsin. Schematic view of the bovine rhodopsin highlighting the visual opsins spectral tuning sites ([19, 20]): *OPN1lw* (red), *RH2* (green), *OPN1sw2* (blue), *OPN1sw1* (violet) and *RH1* (black). Shared spectral tuning sites are indicated by shared colours. Residues highlighted in red experienced positive selection in mammals. The bar plot depicts the amino acid composition of the *OPN1sw1* 93 spectral tuning site accounting for the nocturnal and diurnal species and the eutherian orders (or infraclass in the case of marsupials) in which these amino acids were found

We implemented site-specific selection tests based on the ω-ratio (the ratio between the non-synonymous and synonymous rate of substitution, ω = *dN*/*dS*) to assess the selective pressures acting on the mammalian opsins [17, 18]. *OPN1sw1*, *OPN4m*, *OPN3* and *RRH* showed evidence of site-specific positive selection, while *RH1* showed evidence of site-specific negative selection (Additional file 1: Table S1). In addition, the *OPN1sw1*-conopsin 50I, 93P, 100 N, 108H, 314Q and 334 T residues (human amino acid residues, based on bovine rhodopsin numbering) were positively selected (Bayes empirical Bayes >0.75; Fig. 2). Site 93 has been shown experimentally to be involved in the *OPN1sw1* spectral tuning [19, 20]. Further analysis demonstrated that the amino acid composition at site 93 segregates with the nocturnal/diurnal activity patterns of mammals (bar plot of Fig. 2), as T, S, A and M amino acids were mostly associated with nocturnal lineages, while P, V and C were with diurnal.

To assess the prevailing selective pressures acting at the branch level in the mammalian opsins, we performed branch-specific selection analysis. The branch selection models were implemented in two phases: computing the free-ratios model to calculate the opsins adaptive trees (ω-trees) and test ing the one-ratio vs. two-ratio hypothesis to validate lineage-specific adaptive events [21]. The species-specific evolutionary rates (ω-lineages), corresponding to the sum of the root-to-tip ω-branches in each of the opsin ω-trees (i.e. a ω-tree linearization; Additional file 2: Table S2) were analysed and suggested outlier tendencies among Monotremata, Marsupialia, Carnivora, Chriroptera, Artiodactyla and Primata (Additional file 3: Figure S1). The root-to-tip procedure was particularly effective recovering ω-ratio variations in the terminal branches and clades of the mammalian tree: we predict that this pattern is due to the tree-linearization, which might dilute ω-lineages tendencies in the internal branches. Thus, we proceed with these results by focusing on the most-recent adaptations of mammals. Further analyses of these lineages were carried out by applying the one-ratio vs. two-ratios branch-specific test and significant results were obtained (*p*-value <0.002; Bonferroni corrected for 24 tests) in the terminal lineages of platypus (*Ornitorhynchus anatinus*, *RRH*, Likelihood ratio test (LRT) = 11.849; *OPN4m*, LRT = 19.186), bearded seal (*Erignathus barbatus*, *RH1*, LRT = 10.312) and Sowerby’s beaked whale (*Mesoplodon bidens*, *RH1*, LRT = 29.651) (Additional file 4: Table S3).

### Ancestral character reconstructions

In order to retrace the photic evolution of the mammalian eye, ancestral reconstructions were carried out in the emerging nodes of mammals, monotremes, therians, marsupials and placentals for four photic-related characters [activity pattern, violet/ultra-violet sensitive (VS/UVS) vision, orbit convergence and visual acuity].

**Fig. 3:**
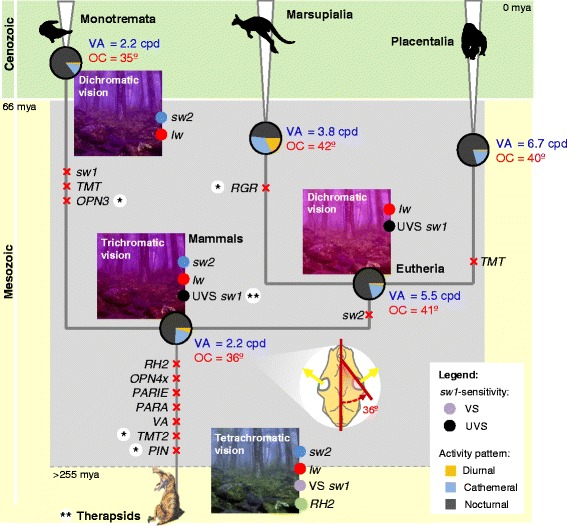
Evolution of opsins and photic-related characters in mammals. Schematic view of the global nocturnal bottleneck hypothesis, linking both the results from the synteny and the ancestral reconstruction analyses: a nocturnal period (represented in grey) is assumed to affect both the mammalian ancestor and the emerging lineages. Global and lineage-specific losses of opsins are indicated with a red cross; opsin losses that were not supported by a consistent synteny are marked with an asterisk (*). Ancestral reconstructions of the activity pattern are represented in pie charts, each slice representing the probability of each state (nocturnal, diurnal and cathemeral). Ancestral reconstructions of the violet and ultra-violet sensitive (VS and UVS) vision are represented by a violet or a black circle, respectively, near the *OPN1sw1* opsin (*sw1* was used to perform the inferences). Uncertainties about the opsin presence/absence are marked with a double asterisk (**): UVS vision in the mammalian ancestor node is only hypothetical (it could not be determined because the *sw1* is not present in monotremes); the therapsid opsin profile is not known since genetic data is not available for therapsids. The potential vision of ancestral mammals was inferred considering both the conopsin content of each node and their spectral sensitivities [blue for *OPN1sw2* (*sw2*), red for *OPN1lw* (*lw*) and saturated pink for UV *sw1*]. Ancestral inferences of the orbit convergence (OC, degrees) and visual acuity (VA, cycles per degree) are indicated in the corresponding nodes. A reconstruction of a skull with an orbital convergence angle of 46° (which correspond to the inferred angle in the node of mammals) is included. The Shennongjia virgin forest (https://commons.wikimedia.org/wiki/File:Shennongjia_virgin_forest.jpg) and the *Pristerognathus* (https://commons.wikimedia.org/wiki/File:Pristeroognathus_DB.jpg) are licensed under the Attribution-ShareAlike 3.0 Unported license (the license terms can be found on the following link: https://creativecommons.org/licenses/by-sa/3.0/)

Ancestral reconstructions for the mammalian nocturnal, cathemeral and diurnal activity patterns and VS/UVS vision were implemented using the *RH1* and *OPN1sw1* ω-trees, respectively (Additional file 2: Table S2). Previous studies validated the use of these opsins as genetic markers in making ancestral inferences, such as the *RH1* mediating the phototransduction in low-light levels, which has been related with the mammalian activity pattern evolution [22–26], whereas sequence variations in the *OPN1sw1* determine spectral tuning changes in the violet/ultra-violet range [27–30]. The all-rates-different time-continuous Markov model adequately fitted the evolution of activity pattern (LRT = 9.743, *p*-value = 0.002) and VS/UVS vision (LRT = 8.050, *p*-value = 0.004 in mammals. The maximum likelihood ancestral inferences suggested a nocturnal lifestyle (i.e. nocturnal state with higher probability) in the ancestral nodes of mammals, monotremes, therians, marsupials and placentals (0.743, 0.859, 0.791, 0.482 and 0.794 respectively; pie charts in Fig. 3 and Additional file 5: Figure S2). Ancestral reconstructions of VS/UVS vision strongly supported (with probability 1.000) the UVS condition in the ancestral therians, marsupials and placentals (*sw1* sensitivity in Fig. 3; Additional file 5: Figure S2). *OPN1sw1* is not present in monotremes. Therefore, the VS/UVS inference in the ancestral node of mammals could not be determined.

The ancestral character reconstructions were also performed for the orbital convergence and visual acuity. Orbit convergence (degrees, °) measures the orientation of the orbit bone relative to the midsagittal plane [8, 9, 31]; visual acuity (cycle per degree, cpd) is a measure of the resolution capacity of the visual system [1]. Both orbit convergence and visual acuity were primarily tested for phylogenetic autocorrelation and were significantly dependent on the opsin ω-trees (Moran’s I test, *p*-values <0.003, Bonferroni corrected for 16 comparisons; Additional file 6: Table S4), demonstrating that opsin genes are appropriate genetic markers to perform ancestral inferences for these characters. The maximum likelihood inferences, obtained employing the Brownian motion model in the opsin ω-trees, indicated low orbit convergence (< 42°, i.e. panoramic vision; the orbit orientation can be used as a surrogate variable for the degree of panoramic/binocular vision [9]) and a low visual acuity (< 7 cpd) in all the studied nodes (mammals, monotremes, eutherians, marsupials and placentals; Additional file 5: Figure S2, Additional file 6: Table S4 and Fig. 3). The inferred phenotypes are similar to the orbit disposition and visual acuity observed in the nocturnal brown rat (32° and 1.6 cpd, respectively) but contrast with humans, which have high orbit convergence and visual acuity (79.3° and 64 cpd, respectively) [32–34]. Furthermore, we found a significant association between the orbit convergence and the nocturnal/diurnal activity patterns of extant mammals, suggesting that nocturnal mammals generally have divergent orbits while diurnal mammals possess convergent orbits (*p*-value = 0.0076, Wilcoxon test, Additional file 7: Figure S3).

Since branch-site tests of positive selection can retrieve misleading results [26], our study considered both branch- and site-specific selection tests (the branch-site tests have been then discarded from the analyses). Moreover, to increase the confidence of our results: (*i*) we have considered only the sites under positive selection that have been reported as having a role in spectral tuning (in our case, the site 93 of the *OPN1sw1*); (*ii)* the branch-selection tests were Bonferroni corrected avoiding false positives; (*iii*) the ancestral inferences for the discrete characters (activity pattern and UVS/VS vision) were only used with the opsin ω-trees with known role in these processes (i.e. *RH1* and *OPN1sw1*, respectively); and (*iv*) the inferences for the continuous characters (visual acuity and orbit convergence) were firstly tested for phylogenetic signal.

## Discussion

### Opsin gene content in ancestral mammals

Based on genomic analyses, we detected that the *RH2*, *VA*, *PARA*, *PARIE* and *OPN4x* (and possibly *PIN* and *TMT2*) opsins were absent in mammals, suggesting that ancestral mammals evolved with a reduced number of opsins. The extensive loss of opsins from the mammalian genomes indicates that early mammals should have inhabited environments where relevant photic stimuli were absent, permitting the corresponding opsins to become pseudogenized with none (or minor) effect on species fitness. A nocturnal phase (or a progressively nocturnal phase) in the early mammals would be concordant with the loss of the *RH2*, *VA*, *PARA*, *PARIE* and *OPN4x* opsins.

The *RH2* photoreceptor responds to the green range of the light spectrum [12] and its loss represented a decrease in the ability of mammals to discriminate colours. While the *RH2* loss does not imply that colour vision was compromised in ancestral mammals since they most probably had a trichromatic visual system with the *OPN1sw1*, *OPN1sw2* and *OPN1lw* conopsins, it is suggestive that visual acuity was being reduced, which constitutes a plausible adaptation to scotopic or mesopic environments. Moreover, the *RH2* pseudogenization was reported in other nocturnal species, as the barn owl, indicating that the loss of the RH2 pigment must be common in scotopic environments [35].

The loss of the *OPN4x* opsin suggests that mammals have simplified their circadian responses. However, they were not compromised because the *m*-type melanopsin (*OPN4m*) is still present in the mammalian retina. Melanopsins are expressed in a particular group of retinal cells localized in the ganglion cell layer, where they performed non-image forming tasks [14]. Notably, this cell layer was reported to be very reduced in the nocturnal-type retina [1] which could have potentiated the loss of the *OPN4x* in mammals. Nevertheless, further studies in the melanopsin gene family are still necessary to understand the consequences of losing *OPN4x* and maintaining *OPN4m* in the circadian response. The same applies to the *TMT* gene family, for which a possible loss was reported (*TMT2*): not only the *TMT* opsins have an undifferentiated expression (both were reported to be expressed in the eyes, brain and other internal organs of vertebrates) but also their photoreceptive roles remain, particularly for Tetrapoda, unknown [4].

We have evidence that all the pineal opsins (*PARA*, *PARIE*, *VA* and *PIN*) were lost from the mammalian genomes. Pineal opsins are expressed in the third eye of vertebrates, which is responsible for the regulation of circadian rhythms and hormone production for thermoregulation [4]. The mammalian third eye, in contrast with other vertebrates, lacks a parietal organ and has a pineal organ with secretory functions only (pineal gland) [36]. We propose that the extensive loss of pineal opsins is correlated with the simplification of the third eye in mammals due to their nocturnal emergence. In nocturnal environments, which are less energetic environments, nocturnal mammals have to develop new thermoregulation strategies to become less reliant on external sources of energy to maintain body temperature: indeed, in contrast with their ancestors, mammals are endothermic [37, 38]. Thus, we propose that the third eye, which most certainly had a role in the ectothermic response [39], partly degenerated when mammals evolved to depend on endothermic metabolism, leading to the loss of the pineal opsins. Likewise, the loss of two pineal opsins (*PARA* and *PARIE*) has also been reported in birds, which like mammals, are endothermic [35].

The syntenic analyses showed global (*RH2*, *OPN4x*, *TMT2*, *PIN* and *VA* and possibly *PIN* and *TMT2* were all absent in mammals) but also lineage-specific losses of opsins among mammals (monotremes were the only lineage with *OPN1sw2*; *OPN1sw1* and *OPN3* were present only in therians; *TMT* was only present in marsupials; the *RGR* gene was absent in marsupials) thus suggesting that the nocturnal period continued after the mammalian lineages diverged, affecting both the most recent common ancestor of mammals [215.5 million years ago (mya), which corresponds with the emergence of mammals [40]] and the earliest emerging lineages. Hence, we propose a mesopic-to-scotopic bottleneck: in an initial mesopic period the *RH2*, *OPN4x*, *TMT2*, *PIN* and *VA* were lost, but *OPN1sw1*, *OPN1sw2* and *TMT* were retained, and subsequently differentially segregated among monotremes, marsupials and placentals in a scotopic period. Indeed, the progressive decrease of visual acuity, expected by the initial loss of *RH2* in all mammals and then the loss of *OPN1sw1* in monotremes and *OPN1sw2* in therians, suggests that mammals went through photic environments with progressively lower luminance levels.

Recent evidence revealed that the majority of therapsids were mesopic or scotopic [41], suggesting that the occupation of nocturnal niches may have started before the divergence of therapsids and mammals. However, the same study also showed that the nocturnal activity appeared early in the synapsid history, evolving independently several times [41]. Without knowing which were the opsin syntenic patterns in therapsids, we can only advance that the beginning of the nocturnal bottleneck was before the emergence of mammals, i.e. > > 215.5 mya. While the beginning of the nocturnal bottleneck cannot be stated precisely, the end of the nocturnal bottleneck is generally agreed to have occurred during the Cretaceous/Paleogene boundary (66 mya), in which the mass extinction of the large reptiles, provided mammals with the opportunity to occupy diurnal niches [38, 42]. In agreement, the predominant diurnal mammalian orders were shown to evolve at <66 mya (anthropoid primates, artiodactyls and perissodactyls evolved at 31.3, 65.4 and 57.0 mya [40], respectively).

### Scotopic and UVS vision in ancestral mammals

In this study, we found evidence that *RH1* evolved under purifying selection and associated with the activity pattern of mammals, suggesting they had a nocturnal lifestyle for most of their evolutionary history. It is expected that a nocturnal lifestyle would rely on the role of the rhodopsin (*RH1*), which is expressed in the rods that are photoreceptive cells activated at low luminance levels [5, 6]. Thus, we advance that the preservation of the *RH1* functions relates to the retention of the rods in the mammalian retina (which is rod-dominated) to guarantee dim-light photic responses at higher levels in the brain (e.g. increased visual sensitivity) [43]. A completely different scenario of *RH1* adaptive evolution was observed in birds, which evolved with evidence of site-specific positive selection [35]. Birds, distinct from mammals, are more-highly visual, and with the exception of some specific lineages (e.g. strigiformes and apterygiformes), generally occupy diurnal niches [35]. Thus, the selective signatures on the *RH1* gene appear to correlate with nocturnal/diurnal lifestyles. Recent evidence suggests that the conservative evolution of the *NRL* eye development gene in mammals is associated with the augment of rod photoreceptors in the mammalian retina [44]. These results provide evidence of an evo-devo mechanism for the activity pattern evolution in mammals and birds.

Evidence of diversifying selection was detected in the *OPN1sw1* opsin, particularly in the 93 spectral tuning site, indicating that mammals evolved adaptive strategies that included the retuning of the *OPN1sw1* sensitivity. Site 93 has been previously reported to be involved in the *OPN1sw1* tuning: the P93T substitution significantly shifts the *OPN1sw1* into the UVS in aye-aye primate [19], and also it is involved in synergistic effects with other sites (46, 49, 52, 81, 86, 114 and 118) [20]. We observed that the amino acid variability at site 93 was related to the activity pattern of mammals, where the 93 T (which provides UVS) is mostly associated with nocturnal lineages and the 93P (which provides VS) with diurnal. The scotopic/UVS and photopic/VS associations were expected since many nocturnal mammals have lost mechanisms of ultra-violet-blocking and are ultra-violet-sensitive [38]. In addition, our findings are congruent with Hunt et al. (2009) who stated that the *OPN1sw1* opsin was firstly adapted to respond to ultra-violet light and later evolved to become more sensitive to light within the violet range [5]: we could infer that ancestral mammals, including the most recent common ancestor of eutherians, placentals and marsupials (and for which we have inferred a nocturnal lifestyle) possessed UVS vision. Evolving a UVS *OPN1sw1* in nocturnal environments suggests ancestral mammals would benefit from having this sensitivity while in dim-light; however, a photo-biological reason for such adaptation remains elusive. Maybe, ancestral mammals would benefit from the ultraviolet vision during the twilight periods of the day, which would provide the necessary luminance for the activation of the sw1 pigment [45].

### Ecology of the mammalian ancestral eye

Ancestral character reconstructions showed that ancestral mammals (including the early monotremes, therians, marsupials and placentals) possessed low visual acuity and low orbit convergence. The inferred phenotypes are compatible with the general conformation of the extant nocturnal mammals.

Evidence of lower visual acuity in early mammals supports a scotopic-adapted retina. Indeed, in nocturnal environments it is more important to maximize the amount of light one can capture, regardless of being able to distinguish among spectral wavelengths [7, 8]. Veilleux et al. (2014) demonstrated that lower visual acuity is associated with nocturnal species [34]; in addition, it has been shown that adaptations which enhance visual sensitivity in low-light are generally incompatible with high acuity [1, 46]. This is partially from the differentiated number of rods (more sensitive to small quantities of light; more common in scotopic retinas) and cones (less sensitive but more accurate for detail, i.e. better performance distinguishing between different colours; more common in photopic retinas) in scotopic and photopic adapted retinas ([43, 47] and reviewed in [48]). Thus, lower visual acuity is associated with rod-dominated retinas specialized for enhancing visual sensitivity, characteristics that we assign to the ancestral mammals. Two pieces of evidence corroborate this statement: (*i*) the preponderance of rods and (*ii*) the absence of the *RH2* opsin in extant mammals [5, 6].

Our analysis also revealed that early mammals had a lateral disposition of their orbits, thus suggesting the possession of panoramic vision. Presumably, nocturnal animals would benefit from a frontal disposition of the orbits (binocularity) because it maximizes the sensitivity to low-light levels by doubling the chance of registering a photon on the visual field [9]. Birds are a clear example: while most of the diurnal birds possess a divergent pattern of orbit disposition, the nocturnal ones (e.g. owls) have frontally placed eyes in order to increase visual sensitivity [49]. In mammals, this tendency appears to be inverted: our results showed that extant nocturnal mammals tend to have panoramic vision. It must be noted that Walls (1942) reported that binocular contrast sensitivity is only slightly more effective than monocular sensitivity in a normal visual system [1], and thus panoramic vision, while not being the optional phenotype one would expect in scotopic environments, does not necessarily imply a decreased visual sensitivity. However, if mammals could have developed strategies that increased visual sensitivity with both binocular and panoramic vision, why did early mammals evolve with a clear pattern of divergent orbits? An alternative hypothesis to explain the role of panoramic vision in ancestral mammals may be related to differences in prey/predator lifestyle. Panoramic visual fields have been associated with taxa subjected to predation (such as artiodactyls, equids and lagomorphs) and often is considered to be an advantage for identifying approaching predators [1, 46]. A divergent configuration of the orbits would provide a wider field of vision and a broader view of the surrounding area, thus simultaneously allowing the detection of photic stimuli from different directions. In addition, it was shown that predators have generally higher visual acuity [34], which decreases the likelihood of ancestral mammals (for which we inferred low visual acuity) being highly-efficient predators. Therefore, we hypothesize that panoramic vision in ancestral mammals facilitated the identification of potential predators. This hypothesis is consistent with both the predation pressures imposed by the successful reptiles during the Mesozoic, and the paleontological evidence, which suggests ancestral mammals were small arboreal animals and most likely, easy prey [50, 51].

### More-recent photic adaptations in mammals

The removal of the predation pressures imposed by the large dinosaurs during the Mesozoic (66 mya), left mammals with the opportunity to explore other photic environments [38, 42]. We showed that three mammalians species (platypus, beard seal and Sowerby’s beaked whale) have undergone more-recent adaptations for some of the studied opsins. The platypus lineage showed conserved adaptive evolution for the *OPN4m* and *RRH* opsins, suggesting it has maintained the same circadian responses as the ancestral mammals. In agreement with this result, and considering the platypus possess several characteristics of a scotopic/mesopic-adapted eye (low visual acuity and a large optic tectum [52]), we suggest that platypus may be an efficient model-organism to study the ancestral mammalian photic system. The *RH1* opsin showed accelerated evolution in the bearded seal and Sowerby’s beaked whale lineages. The bearded seal and Sowerby’s beaked whale species occupy the cold waters of the North Atlantic, where the low luminosity together with the necessity to dive in deep to feed, may have favoured the retuning of *RH1*. Fasick et al. (2000) and Zhao et al. (2009) showed that *RH1* amino acid substitutions and spectral tuning shifts were correlated with foraging depth in marine mammals [22, 53]. Notice that an acceleration of the *RH1* opsins was also found in the North Atlantic right whale (*Eubalaena glacialis*) lineage; however, it was not significant after the Bonferroni correction (*p*-value = 0.0029/0.002, Additional file 4: Table S3).

## Conclusions

The genomic and phylogenetic lines of evidence advanced here provide new insights on the evolution of mammalian eyes: (*i*) equal and differentiated patterns of opsin loss in mammals suggested a nocturnal period that affected both the common and the emerging lineages of mammals; in addition, we advance that ancestral mammals possessed (*ii*) a nocturnal activity pattern, (*iii*) UVS vision and a scotopic-adapted eye with (*iv*) low visual acuity and (*v*) panoramic vision. In sum, we provide conclusive evidence that mammals (and not only eutherians as Walls initially assumed [1]) were scotopic-adapted for most of their evolutionary history, supporting a global nocturnal bottleneck starting at > > 215.5 mya and lasting until 66 mya.

## Methods

### Synteny and phylogenetic analysis

tblastn searches with protein sequences of the *Homo sapiens* and *Gallus gallus* opsins were performed in the NCBI and Ensembl databases [15, 16]. Synteny analyses were performed using both the Ensembl and Genomicus 64.1 databases [16, 54]. Previously published sequences were collected representing the main phylogenetic groups of mammals for each of the studied opsins (Additional file 8: Table S5). A protein-based coding sequence alignment was performed using the Muscle 3.3 algorithm [55] and subsequently further alignment pruning was employed to remove ambiguous and/or gaps-rich sites. The presence of saturation in base substitution for each of the opsin gene alignments was assessed via the Xia et al. (2003) test [56]; we found no evidence of saturation (Additional file 9: Table S6). jModelTest (version 0.1.1) with Akaike Information criterion was used to estimate the most appropriate nucleotide substitution model [57] and the GTR + I + Γ was the most-appropriate model with 95% of confidence for all opsins, with the exception of *RH1* gene, where HKY + I + Γ was the best-fit model.

### Site- and branch-selection analysis

Site-specific and branch-specific codon substitution models of maximum likelihood, based on the ω-statistic, were implemented in PAML [18]. To eliminate possible confounding effects of phylogeny variation and because we aimed to trace gene evolution within a framework of species evolution, the Meredith et al. (2011) mammalian species tree was employed [40]. The site-specific models were tested comparatively using the LRT: M7 (beta) vs. M8 (beta + ω) and M8a (beta + ω = 1) vs. M8 [17]. M7 and M8 assume a beta-distribution for the ω value between 0 and 1 but M8 additionally allows the occurrence of positively-selected sites (ω > 1) [58]. M8a tests the neutral evolution including a class of neutral evolving sites [58]. Whenever the LRT was significant under the M8 model, the Bayes Empirical Bayes method was used to assess the positively-selected sites (accepted at >0.75 posterior probability) [59].

The branch selection models were carried out in two phases: computing the free-ratios model and testing the one-ratio vs. two-ratio hypothesis [21]. The free ratios model was implemented to calculate the opsin ω-tree. The branches with an outlier ω-ratio (> > 10, mostly due to small *dS* estimates) were recalculated as the ratio between the obtained *dN* and the median *dS*. The species-specific evolutionary rates (ω-lineage), corresponding to the sum of the root-to-tip ω-branches in each of the opsin ω-trees (i.e. a ω-tree linearization), were calculated and standardized to identify ω-lineage outlier tendencies. The ω-lineage was calculated using the distRoot(tree,tips) function of the *adephylo* package of the R statistical software [60, 61]. Statistically significant ω-lineage variations in specific branches were tested via LRT using the one-ratio vs. the two-ratio model comparison: the two-ratio model considers an additional ω-branch parameter for the lineage of interest (ω-foreground) [21].

### Photic-related character dataset construction

A dataset of four photic-related characters (Additional file 10: Table S7) was built for the studied mammalian species. Mammalian activity pattern was determined based on PanTHERIA database and Bennie et al. (2014) [62, 63]. Crepuscular and nocturnal activity patterns were both considered as nocturnal. We have used the amino acid composition at site 86 to perform the VS and UVS inferences. Site 86 has a major role in *OPN1sw1* spectral tuning, since substitutions in this site have been shown to result in large absorption maxima shifts [19, 20]. However, site 86 is not ubiquitous in the spectral tuning of the mammalian *OPN1sw1*and its absorption maximum can vary depending on the amino acid composition of other sites (namely 46, 49, 52, 81, 93, 114 and 118), as noted by Hauser et al. (2014) [64]. In order to avoid these caveats, we opted for using the VS/UVS discrete categories which are more stable for amino acid composition, instead of using absorption maxima measures: the 86F residue is associated with UVS (except in primates) while the Y, S, V, C and L with VS (reviewed in [5, 11, 64]); other amino acid compositions were not considered to UVS/VS inference. In addition, mammalian species for which *OPN1sw1* was reported inactive were excluded from the analysis (based on [65–71], Additional file 10: Table S7). Orbit convergence measurements for non-monotremes mammals were obtained in [32, 33]. Orbit convergence measurements for the platypus and echidna monotremes were performed using the high-resolution computed tomography x-ray skulls (Digimorph project [72], www.digimorph.org). Visual acuity data were obtained from Veilleux et al. (2014) [34]. Platypus and opossum visual acuity measures were retrieved from [52, 73].

### Ancestral character reconstruction analysis

Ancestral character reconstructions were performed using the opsin ω-trees and the data matrices in the *ape* package of the R statistical software [61, 74]: ace(data, tree) function with additional arguments. Discrete characters (type = “discrete”) were calculated based on a time-continuous Markovian model comparing the equal-rates (model = “SYM”) and all-rates-different (model = “ARD”) matrices on a LRT test [75]. Ancestral states were estimated using maximum likelihood inference (if type = “discrete”, maximum likelihood inferences are calculated by default). Continuous characters were firstly tested for phylogenetic autocorrelation with the opsin ω-trees using the Moran’s I statistic [76] that was calculated by the Moran.I() command. The significant associations (*p*-value <0.003, Bonferroni corrected for 16 comparisons; Additional file 6: Table S4) were employed to perform inferences in ancestral nodes, using the maximum likelihood method (method = “ML”) and the Brownian motion model (if type = “continuous” is selected in the ace function, the default model is Brownian motion) [77].

## Additional files

Additional file 1: Table S1. Site-selection tests for the mammalian opsins. The logarithm of the model likelihood is represented by lnL, the number of model parameters is represented by np and the LRT is the likelihood ratio test. The accepted site-selection model are indicated with an asterisk (*) when the M7 model of negative selection is statistically significant, or with a double asterisk (**) if the M8 model of positive selection is statically significant. All the LRT comparisons were performed assuming a significance level of 0.05. (PDF 273 kb)

Additional file 2: Table S2. Opsin ω-tree maximum likelihood estimates. The ω-tree was estimated under the assumption of the branch-specific free-ratios model using the Meredith et al. (2011) tree topology. (XLSX 17 kb)

Additional file 3: Figure S1. Species-specific evolutionary rate for mammalian opsins. ω-lineages were standardized subtracting the median and divided by the interquartile range. Coloured circles correspond to the species subjected to branch selection tests and significant results are indicated with an asterisk (*). (PDF 501 kb)

Additional file 4: Table S3. Species-specific branch selection tests. The one-ratio model (H0) was tested against the two-ratios model considering the alternative hypotheses (H1) of verifying differentiated ω-ratio in the indicated branch. lnL is the logarithm of the model likelihood and the LRT is the likelihood ratio test. All the LRT comparisons were performed with 1 degree of freedom. Significant alternative hypothesis are marked with an asterisk (*) considering a Bonferroni corrected *p*-value of 0.002 (24 test comparisons). (PDF 278 kb)

Additional file 5: Figure S2. Phylogenetic character mapping for the ancestral reconstructions. Ancestral reconstructions of the activity pattern and *sw1*-sensitivity are represented in pie charts, each slice representing the probability of each state. Ancestral inferences of the orbit convergence (degrees) and visual acuity (cycles per degree) are represented by circles that change in size and shade of grey according to the character value. The opsin trees were estimated under the assumption of the branch-specific free-ratios model using the Meredith et al. (2011) tree topology. (PDF 1239 kb)

Additional file 6: Table S4. Ancestral character reconstructions of orbit convergence and visual acuity in mammals. The phylogenetic association between each variable and the opsin ω-tree was tested using the Moran’s I hypothesis test (*p*-value <0.003, Bonferroni corrected for 16 comparisons). Ancestral character reconstructions were performed using the Brownian motion model, excluding the cases in which none (*) or only one (**) species was representing the clades of interest (mammalia, monotremata, eutheria, marsupialia, placentalia). Inferences for each opsin (mean and standard deviation) are shown for each opsin gene tree. The last line of the table summarizes the results considering the mean probability distribution. (PDF 435 kb)

Additional file 7: Figure S3. Orbit convergence vs. activity patterns in mammals. Box plots depicting the association between the orbit convergence (degrees, °) and the activity pattern (nocturnal and diurnal) of extant mammals. (PDF 276 kb)

Additional file 8: Table S5. Mammalian opsin sequences. Accession number of the mammalian opsins sequences used for the evolutionary analyses. (XLSX 103 kb)

Additional file 9: Table S6. Saturation analysis. The presence of saturation in base substitution for each of the opsin gene alignment was tested by comparing half of the expected theoretical saturation index when assuming full saturation (Iss.c, critical value) with the observed saturation index (Iss). The absence of substitution saturation is verified when Iss is lower than Iss.c for a significant p-value. (PDF 107 kb)

Additional file 10: Table S7. Dataset of the eco-morphological variables for the studied mammalian species. Variables: activity pattern (nocturnal, cathemeral and diurnal), VS and UVS OPN1sw1 sensitivity, orbit convergence (degrees, °) and visual acuity (cycle per degree, cpd). OPN1sw1 inactive copies were indicated with an asterisk (*) in the column of the sw1 86 site. Data retrieved from the references [5, 11, 65–72]. (XLSX 22 kb)
