# Supplementary material for: Adaptive genomic evolution of opsins reveals that early mammals flourished in nocturnal environments
Source: BMC Genomics. 2018 Feb 5;19:121. doi: 10.1186/s12864-017-4417-8 (PMC5800076; doi:10.1186/s12864-017-4417-8)
Supplement: Supplementary file 1 — Site-selection tests for the mammalian opsins. The logarithm of the model likelihood is represented by lnL, the number of model parameters is represented by np and the LRT is the likelihood ratio test. The accepted site-selection model are indicated with an asterisk (*) when the M7 model of negative selection is statistically significant, or with a double asterisk (**) if the M8 model of positive selection is statically significant. All the LRT comparisons were performed assuming a significance level of 0.05. (PDF 273 kb) [file 12864_2017_4417_MOESM1_ESM.pdf]

Adaptive genomic evolution of opsins reveals that early mammals flourished in nocturnal environments

Rui Borges, Warren E. Johnson, Stephen J. O'Brien, Cidália Gomes, Christopher P. Heesy and Agostinho Antunes

Table S1

Site-selection tests for the mammalian opsins

The logarithm of the model likelihood is represented by lnL, the number of model parameters are represented by np and the LRT is the likelihood ratio test. The accepted site-selection model are indicated with an asterisk (*) when the M7 model of negative selection is statistically significant, or with a double asterisk (**) if the M8 model of positive selection is statically significant. All the LRT comparisons were performed assuming a significance level of 0.05.

**Gene**	**lnL M7**	**np**	**lnL M8**	**np**	**LRT 7-8**	***p*-value**	**lnL M8a**	**np**	**LRT 8a-8**	***p*-value**	**ω**	
RH1	-18060.196	251	-18060.200	253	0.000	1.000	-18056.699	252	0.000	1.000	0.064	*
OPN1sw1	-17626.205	199	-17605.265	201	41.971	0.000	-17607.898	200	5.266	0.022	0.178	**
OPN1lw	-20776.122	253	-20769.910	255	12.424	0.002	-20766.851	254	0.000	1.000	0.135	
OPN3	-14209.085	127	-14171.960	129	74.251	0.000	-14185.161	128	26.402	0.000	0.31	**
RGR	-15640.311	155	-15634.514	157	11.596	0.003	-15633.702	156	0.000	1.000	0.193	
RRH	-16299.456	165	-16286.261	167	26.391	0.000	-16295.62	166	18.717	0.000	0.273	**
OPN5	-11367.748	163	-11360.527	165	14.441	0.001	-11361.359	164	1.664	0.197	0.089	
OPN4m	-26002.747	169	-25972.711	171	60.072	0.000	-25981.877	170	18.333	0.000	0.239	**
