# Supplementary material for: Adaptive genomic evolution of opsins reveals that early mammals flourished in nocturnal environments
Source: BMC Genomics. 2018 Feb 5;19:121. doi: 10.1186/s12864-017-4417-8 (PMC5800076; doi:10.1186/s12864-017-4417-8)

Adaptive genomic evolution of opsins reveals that early mammals flourished in nocturnal environments

Rui Borges, Warren E. Johnson, Stephen J. O'Brien, Cidália Gomes, Christopher P. Heesy and Agostinho Antunes

Figure S1

<u>Species-specific evolutionary rate for mammalian opsins</u>

ω-lineages were standardized subtracting the median and divided by the interquartile range. Coloured circles correspond to the species subjected to branch selection tests and significant results are indicated with an asterisk (*).

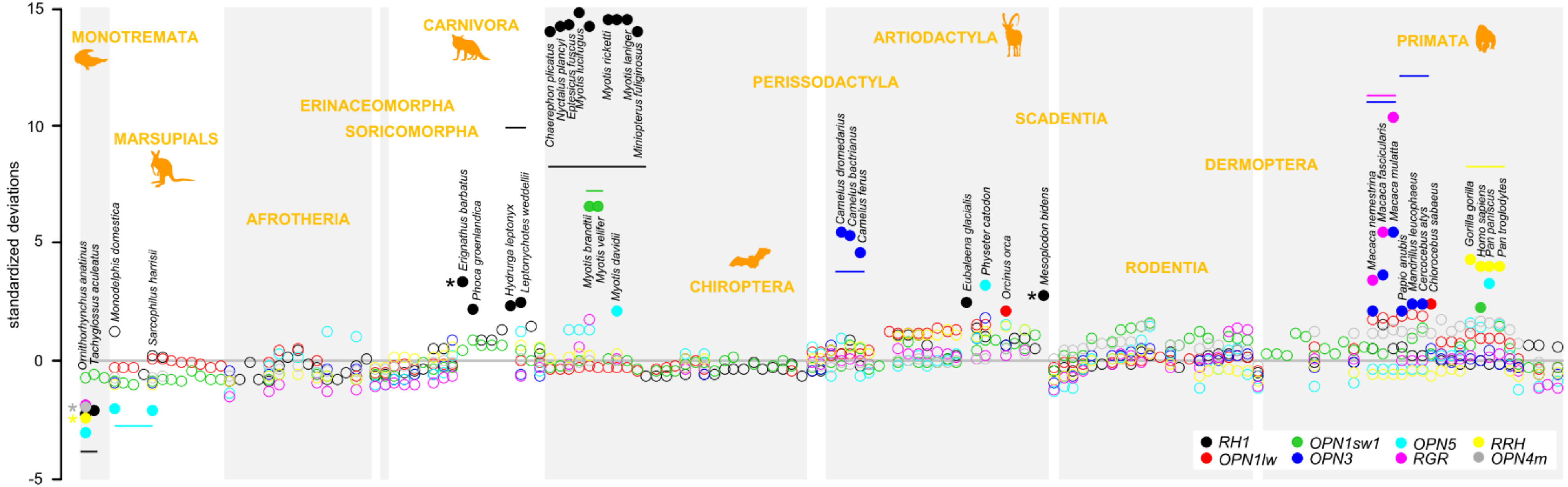

Supplement: Supplementary file 3 — Species-specific evolutionary rate for mammalian opsins. ω-lineages were standardized subtracting the median and divided by the interquartile range. Coloured circles correspond to the species subjected to branch selection tests and significant results are indicated with an asterisk (*). (PDF 501 kb) [file 12864_2017_4417_MOESM3_ESM.pdf]
