# Supplementary material for: Adaptive genomic evolution of opsins reveals that early mammals flourished in nocturnal environments
Source: BMC Genomics. 2018 Feb 5;19:121. doi: 10.1186/s12864-017-4417-8 (PMC5800076; doi:10.1186/s12864-017-4417-8)
Supplement: Supplementary file 4 — Species-specific branch selection tests. The one-ratio model (H0) was tested against the two-ratios model considering the alternative hypotheses (H1) of verifying differentiated ω-ratio in the indicated branch. lnL is the logarithm of the model likelihood and the LRT is the likelihood ratio test. All the LRT comparisons were performed with 1 degree of freedom. Significant alternative hypothesis are marked with an asterisk (*) considering a Bonferroni corrected p-value of 0.002 (24 test comparisons). (PDF 278 kb) [file 12864_2017_4417_MOESM4_ESM.pdf]

Adaptive genomic evolution of opsins reveals that early mammals flourished in nocturnal environments

Rui Borges, Warren E. Johnson, Stephen J. O'Brien, Cidália Gomes, Christopher P. Heesy and Agostinho Antunes

Table S3

Species-specific branch selection tests

The one-ratio model (H0) was tested against the two-ratios model considering the alternative hypotheses (H1) of verifying differentiated ω-ratio in the indicated branch. lnL is the logarithm of the model likelihood and the LRT is the likelihood ratio test. All the LRT comparisons were performed with 1 degree of freedom. Significant alternative hypothesis are marked with an asterisk (*) considering a Bonferroni corrected p-value of 0.002 (24 test comparisons).

Opsin	lnL M0	ω0	Branch	lnL M1	ω1	LRT	*p*-value	
RH1	-18807.123	0.042	*Hydrurga leptonyx* and *Leptonychotes weddellii*	-18803.085	0.687	8.076	0.005	
			Erignathus barbatus	-18801.966	0.856	10.312	0.001	*
			Eubalaena glacialis	-18802.685	0.720	8.876	0.003	
			Mesoplodon bidens	-18792.297	0.778	29.651	0.000	*
			Monotremata	-18806.985	0.000	0.276	0.599	
			Phoca groenlandica	-18803.648	0.414	6.949	0.008	
			Verpertilionoidea	-18807.123	2.160	0.000	1.000	
OPN1sw1	-18224.597	0.14	*Homo sapiens*	-18223.865	0.000	1.464	0.226	
			Myotis brandtii and *Myotis velifer*	-18224.597	33.170	0.001	0.978	
OPN1lw	-21567.983	0.108	*Chlorocebus sabaeus*	-21565.032	0.000	5.902	0.015	
			Orcinus orca	-21567.63	0.000	0.707	0.401	
OPN3	-14446.046	0.263	*Camelus*	-14444.441	999.999	3.211	0.073	
			Macaca sp.	-14446.046	0.084	0.000	1.000	
			Cercopithecinae	-14446.046	0.093	0.000	1.000	
OPN5			Marsupials	-11603.606	0.103	0.393	0.531	
			Myotis davidii	-11603.311	0.201	0.984	0.321	
			Pan sp.	-11603.192	0.417	1.222	0.269	
			Physeter catodon	-11600.687	0.639	6.232	0.013	
			Ornitorhynchus anatinus	-11600.995	0.000	5.616	0.018	
RGR	-16107.05	0.161	*Macaca sp.*	-16105.609	0.000	2.882	0.090	
		0.161	*Ornitorhynchus anatinus*	-16105.468	0.000	3.166	0.075	
RRH	-17046.677	0.237	*Ornitorhynchus anatinus*	-17040.753	0.094	11.849	0.001	*
			Hominidae	-17046.677	46.617	0.000	0.983	
OPN4m	-27303.281	0.166	*Ornitorhynchus anatinus*	-27293.688	0.000	19.186	0.000	*
