# Supplementary material for: Adaptive genomic evolution of opsins reveals that early mammals flourished in nocturnal environments
Source: BMC Genomics. 2018 Feb 5;19:121. doi: 10.1186/s12864-017-4417-8 (PMC5800076; doi:10.1186/s12864-017-4417-8)

Adaptive genomic evolution of opsins reveals that early mammals flourished in nocturnal environments

Rui Borges, Warren E. Johnson, Stephen J. O'Brien, Cidália Gomes, Christopher P. Heesy and Agostinho Antunes

Figure S2

Phylogenetic character mapping for the ancestral reconstructions

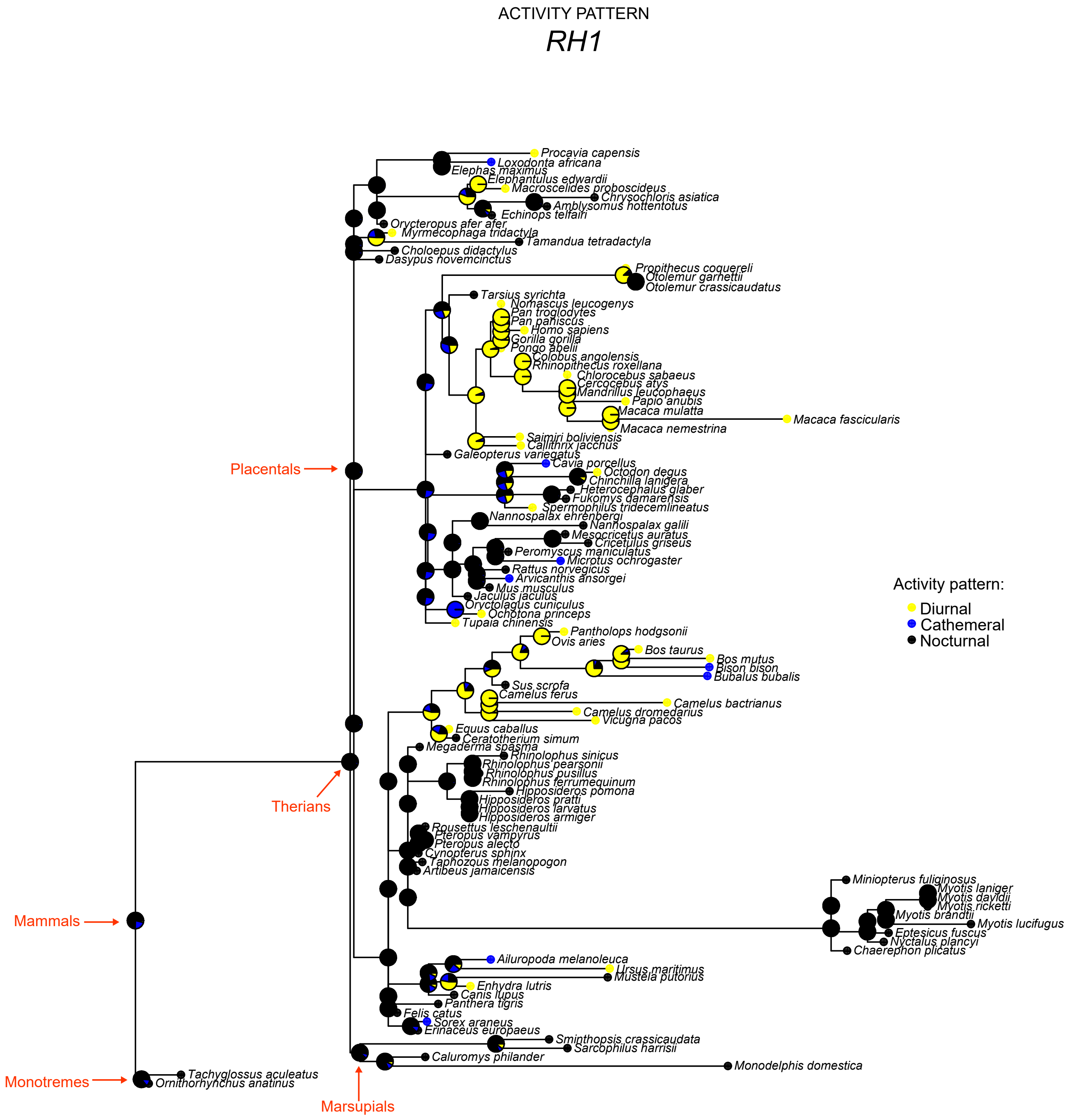

SW1 SENSITIVITY

OPN1sw1

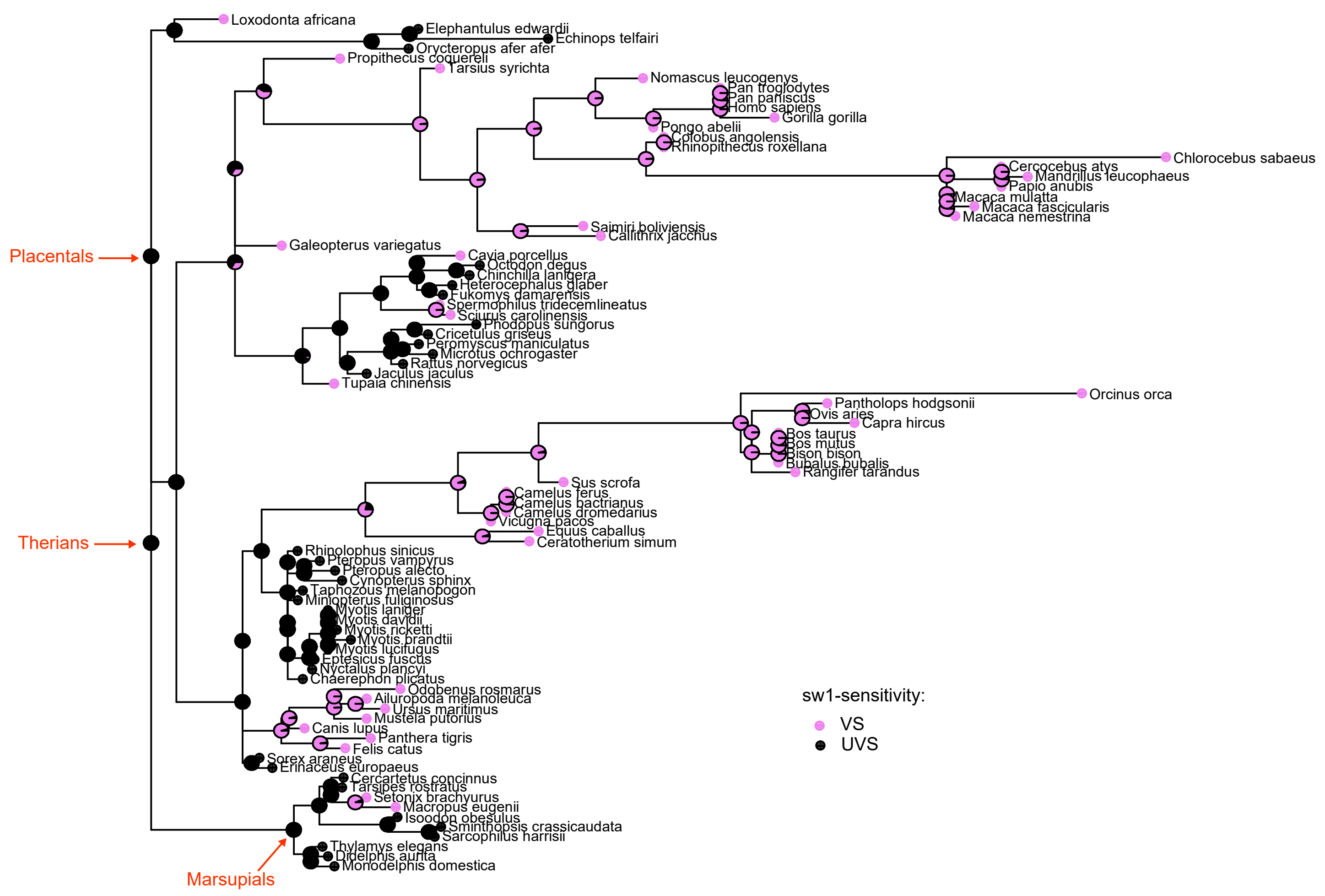

VISUAL ACUITY

RH1

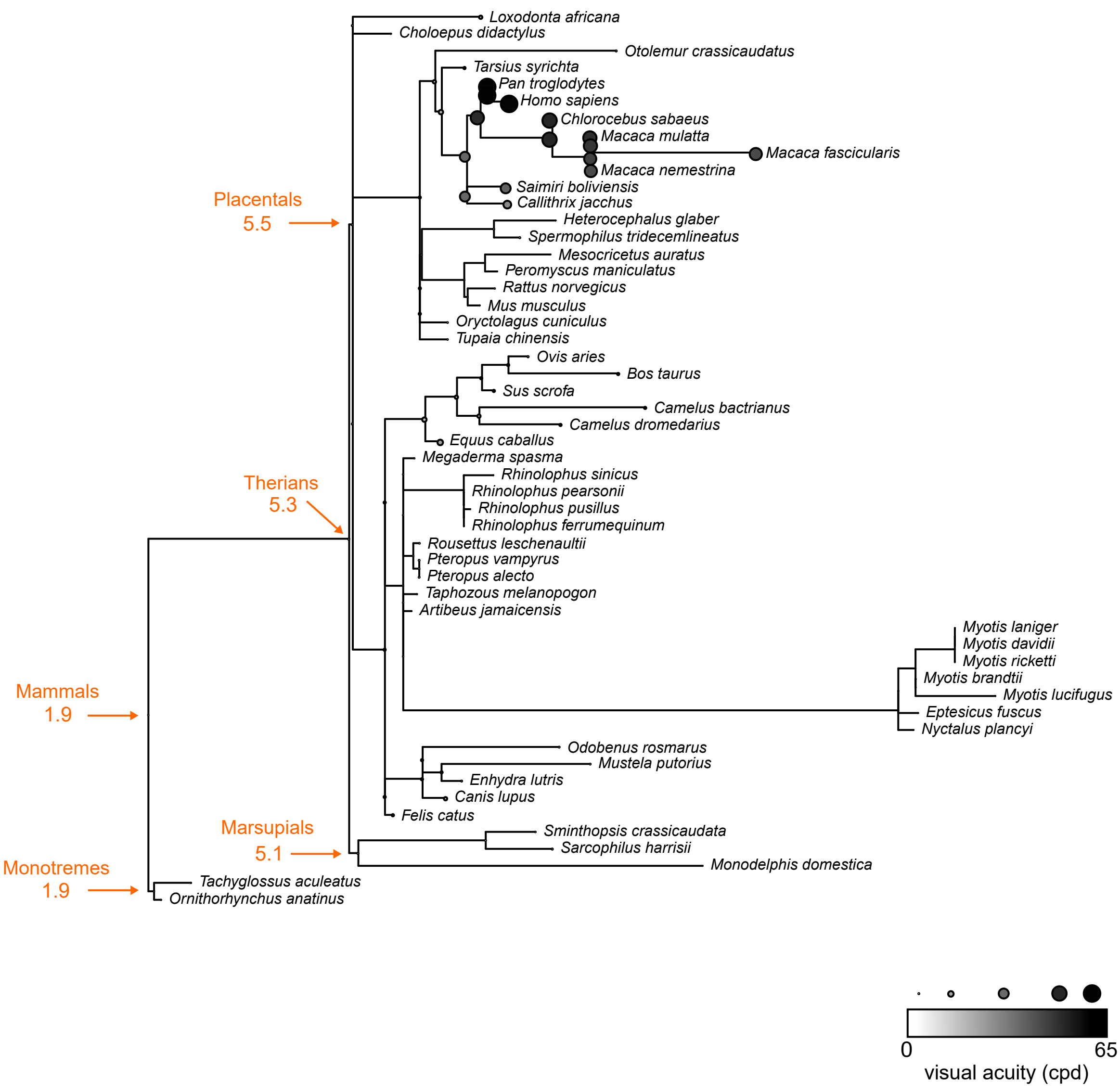

VISUAL ACUITY

OPN1sw1

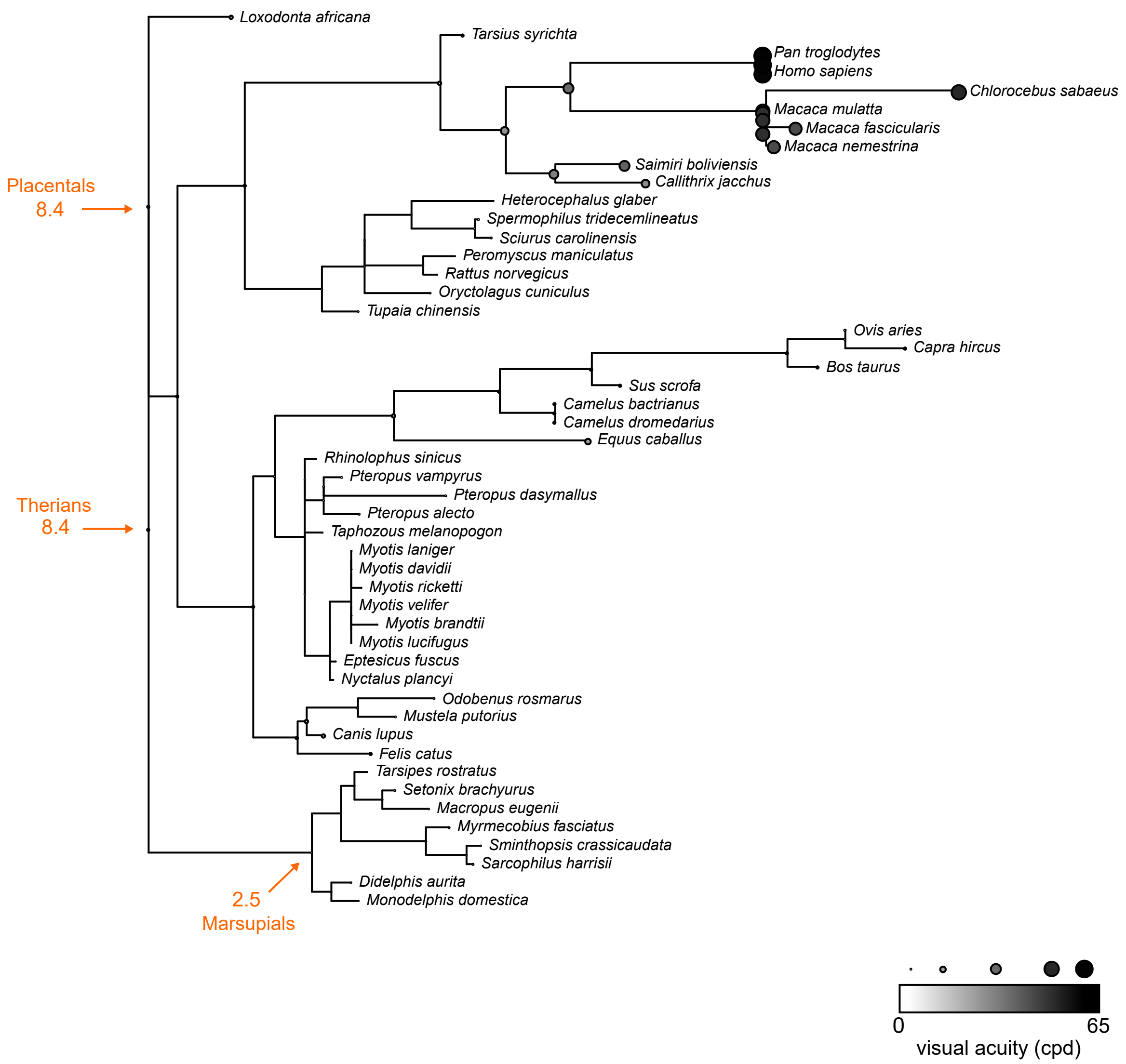

VISUAL ACUITY

OPN1lw

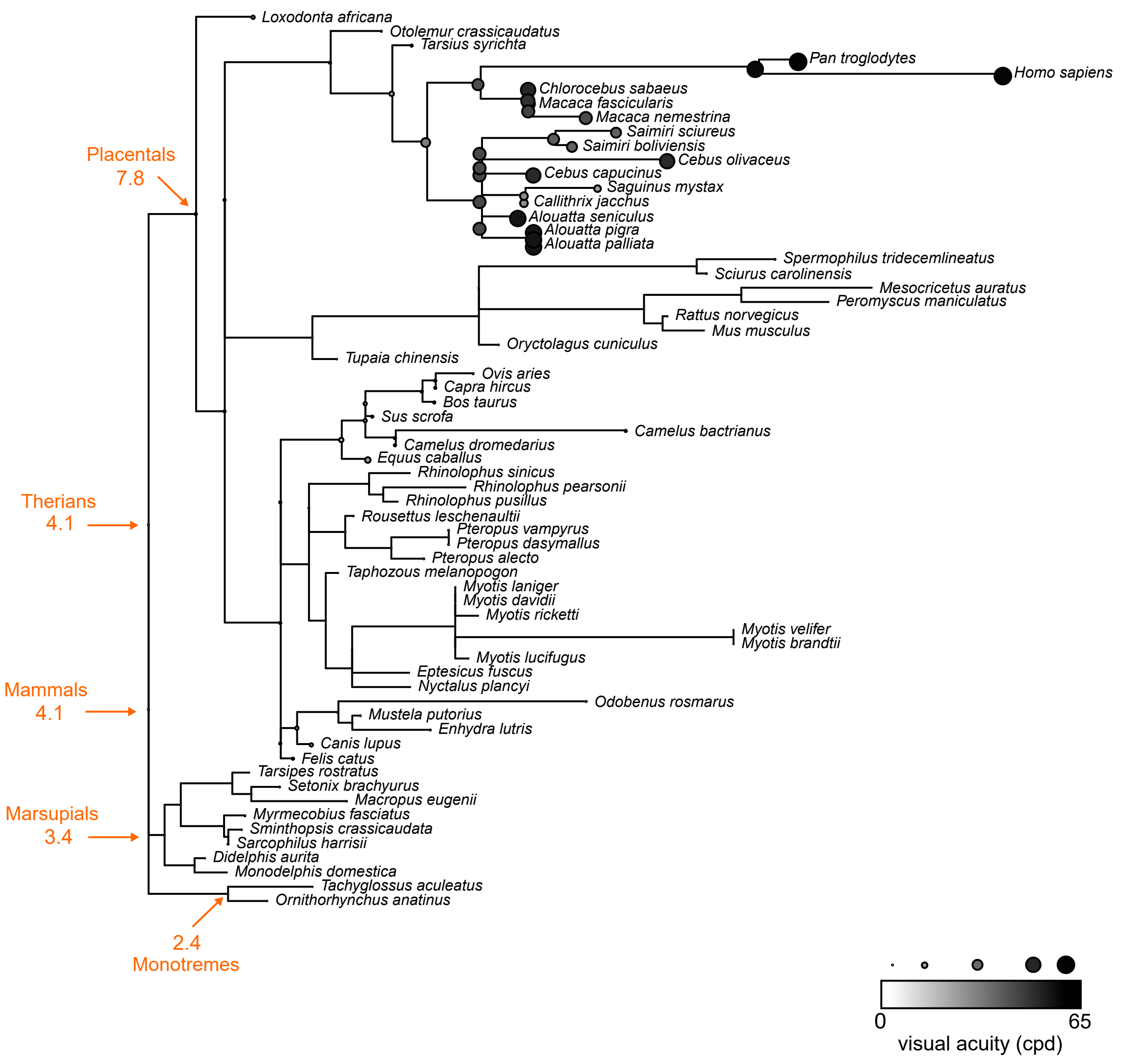

VISUAL ACUITY

OPN3

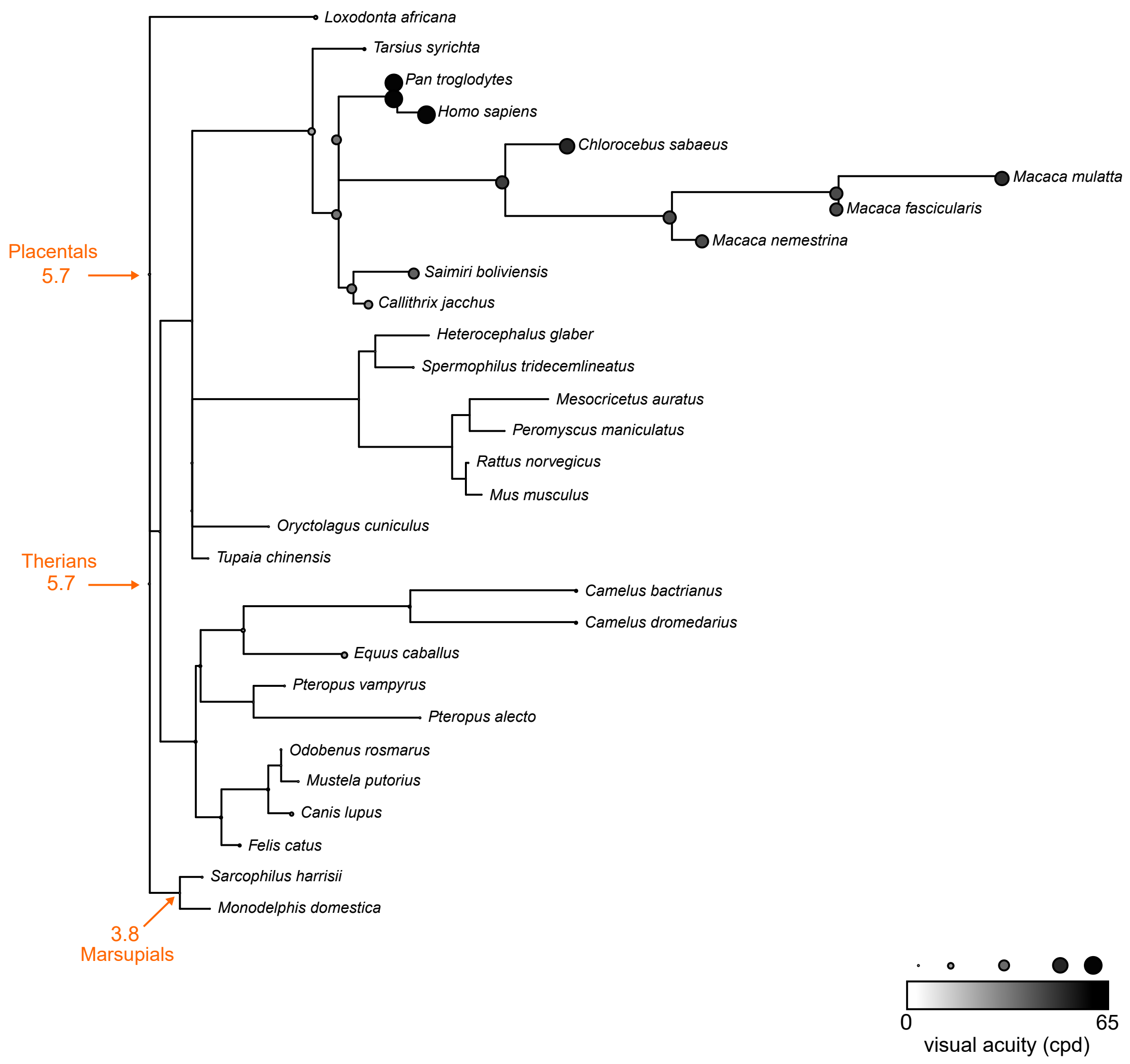

VISUAL ACUITY

OPN5

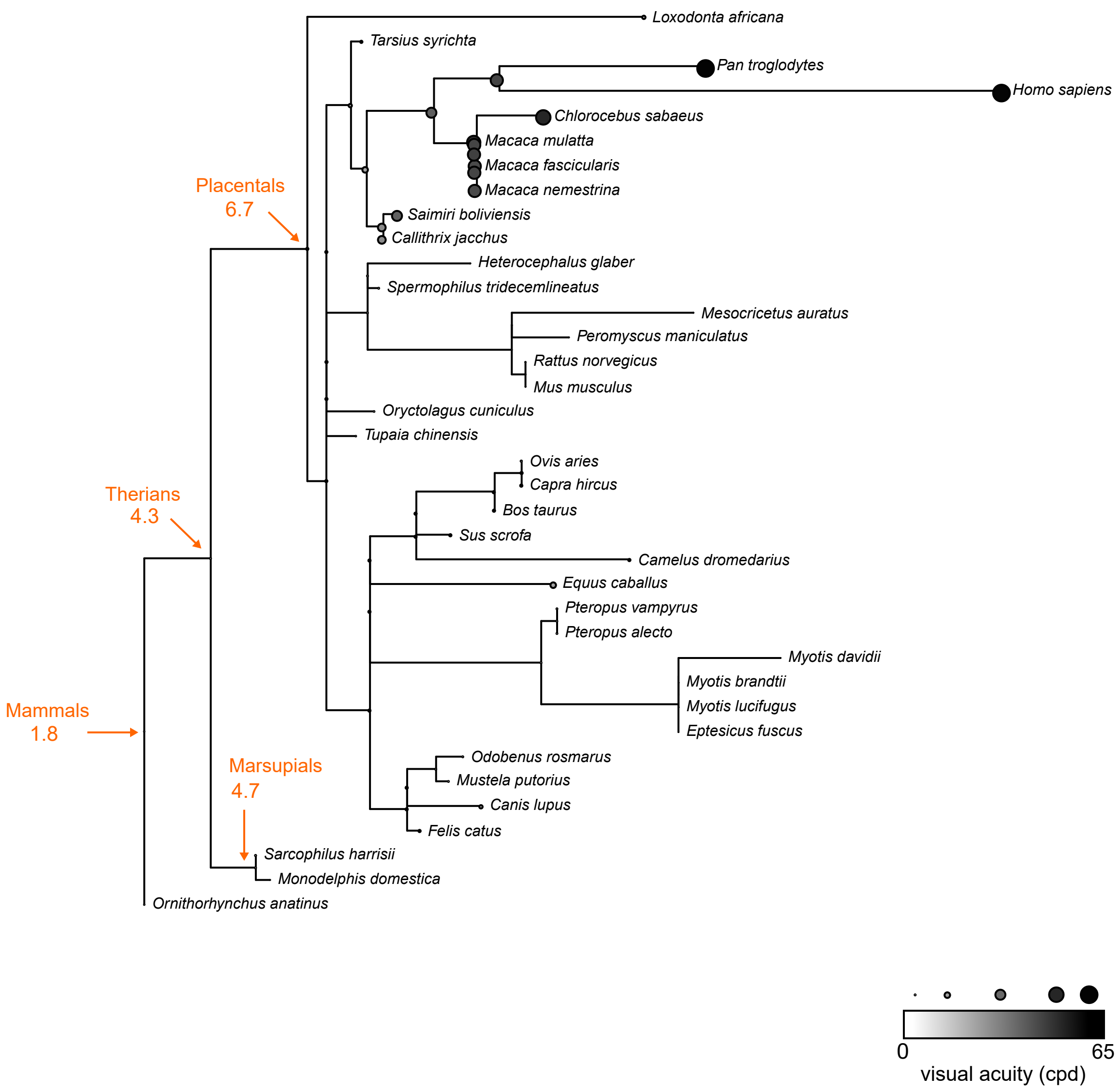

VISUAL ACUITY

RGR

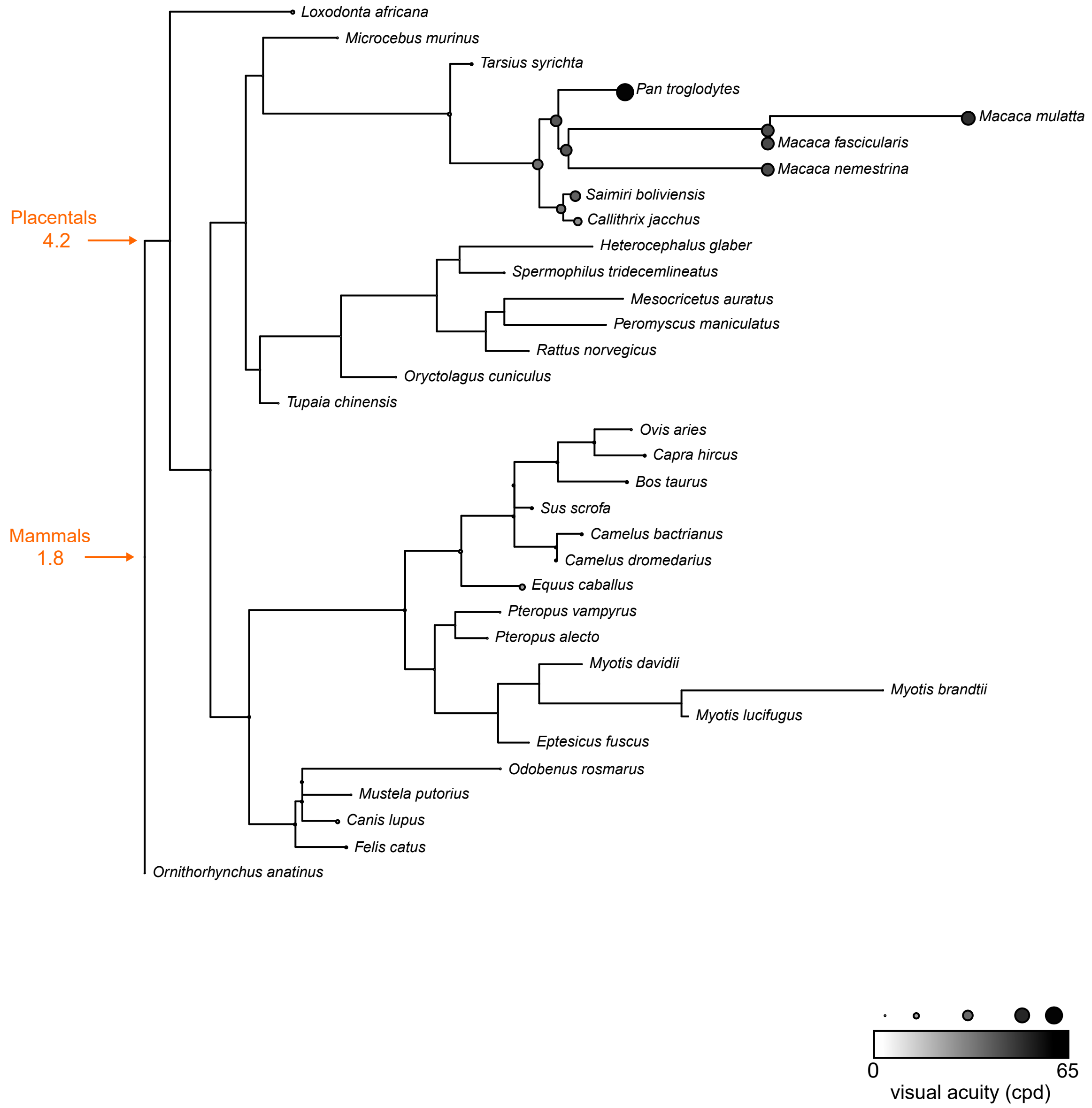

VISUAL ACUITY

RRH

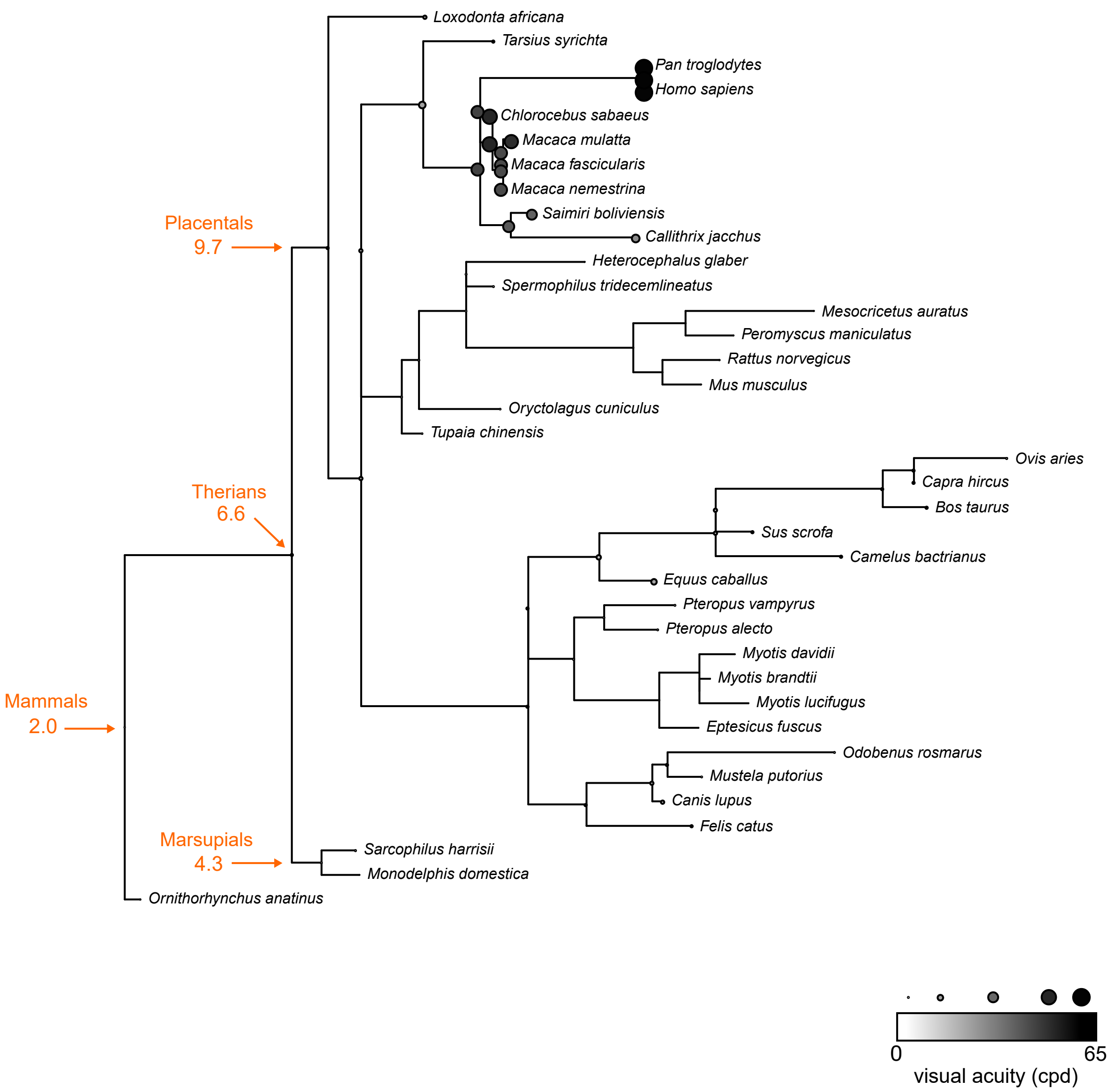

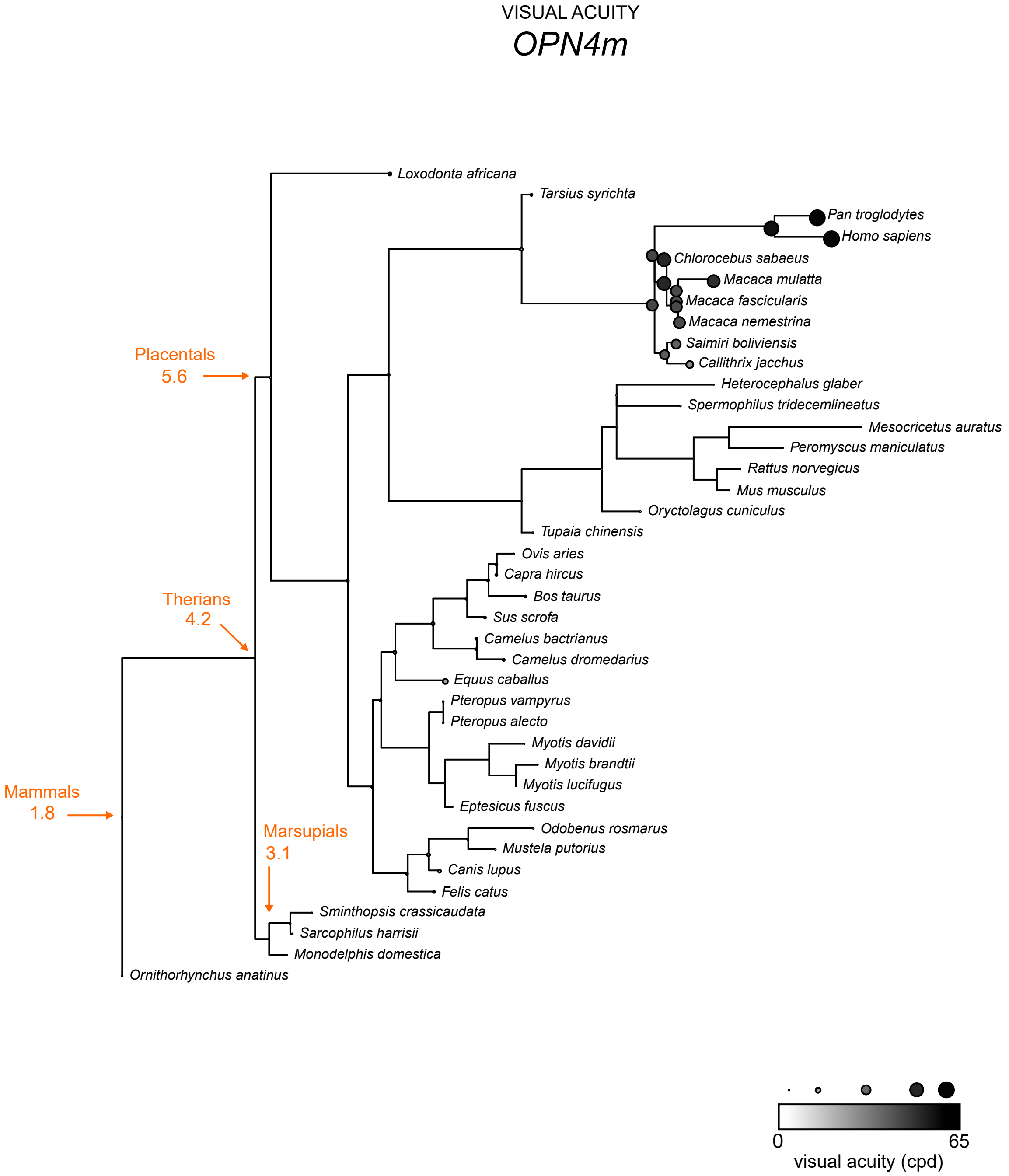
VISUAL ACUITY
OPN4m
Loxodonta africana
Tarsius syrichta
Pan troglodytes
Homo sapiens
Chlorocebus sabaeus
Macaca mulatta
Macaca fascicularis
Macaca nemestrina
Saimiri boliviensis
Callithrix jacchus
Heterocephalus glaber
Spermophilus tridecemlineatus
Mesocricetus auratus
Peromyscus maniculatus
Rattus norvegicus
Mus musculus
Oryctolagus cuniculus
Tupaia chinensis
Ovis aries
Capra hircus
Bos taurus
Sus scrofa
Camelus bactrianus
Camelus dromedarius
Equus caballus
Pteropus vampyrus
Pteropus alecto
Myotis davidii
Myotis brandtii
Myotis lucifugus
Eptesicus fuscus
Odobenus rosmarus
Mustela putorius
Canis lupus
Felis catus
Sminthopsis crassicaudata
Sarcophilus harrisii
Monodelphis domestica
Ornithorhynchus anatinus
Placentals
5.6
Therians
4.2
Mammals
1.8
Marsupials
3.1
0
65
visual acuity (cpd)

ORBIT CONVERGENCE

RH1

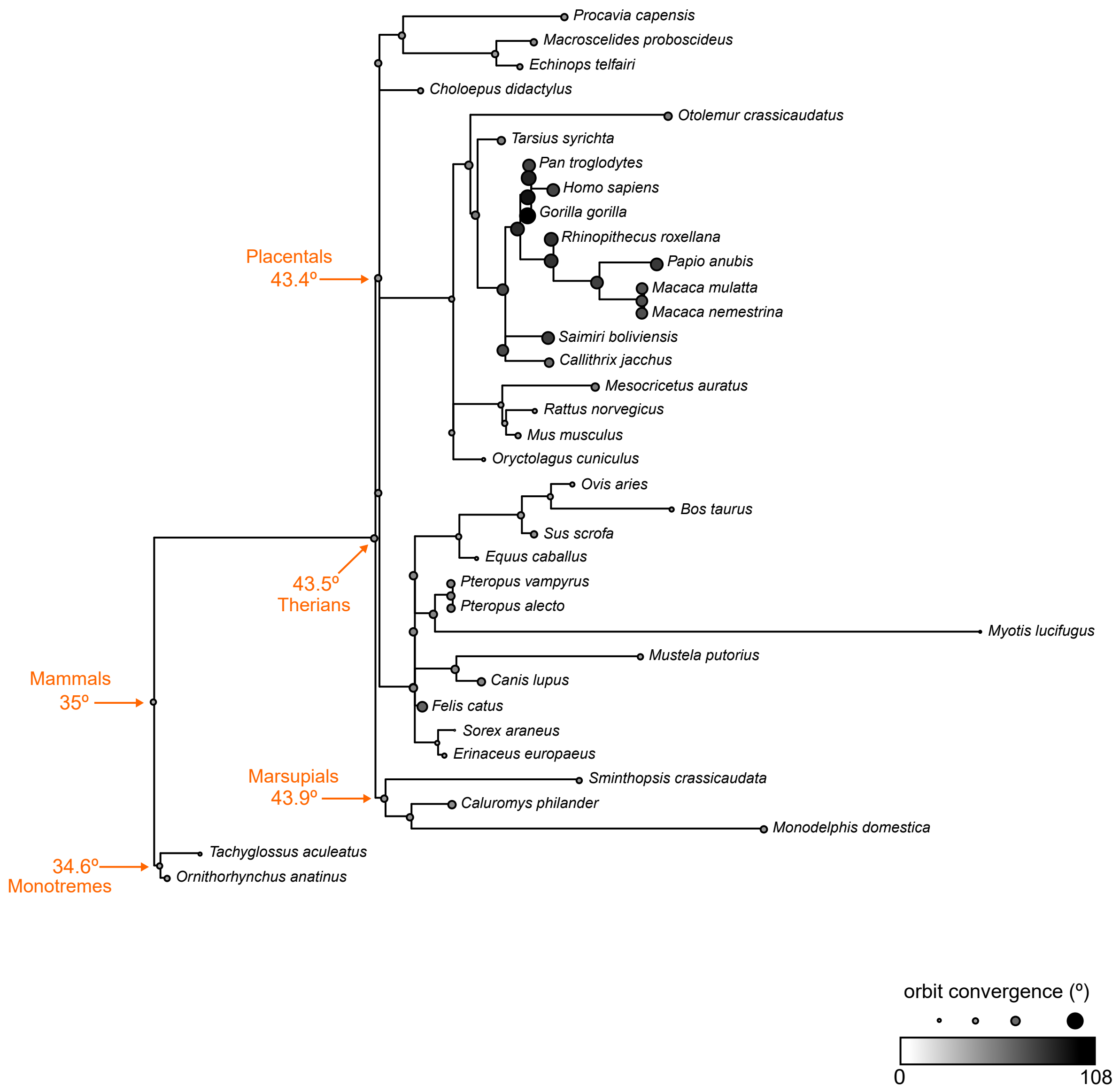

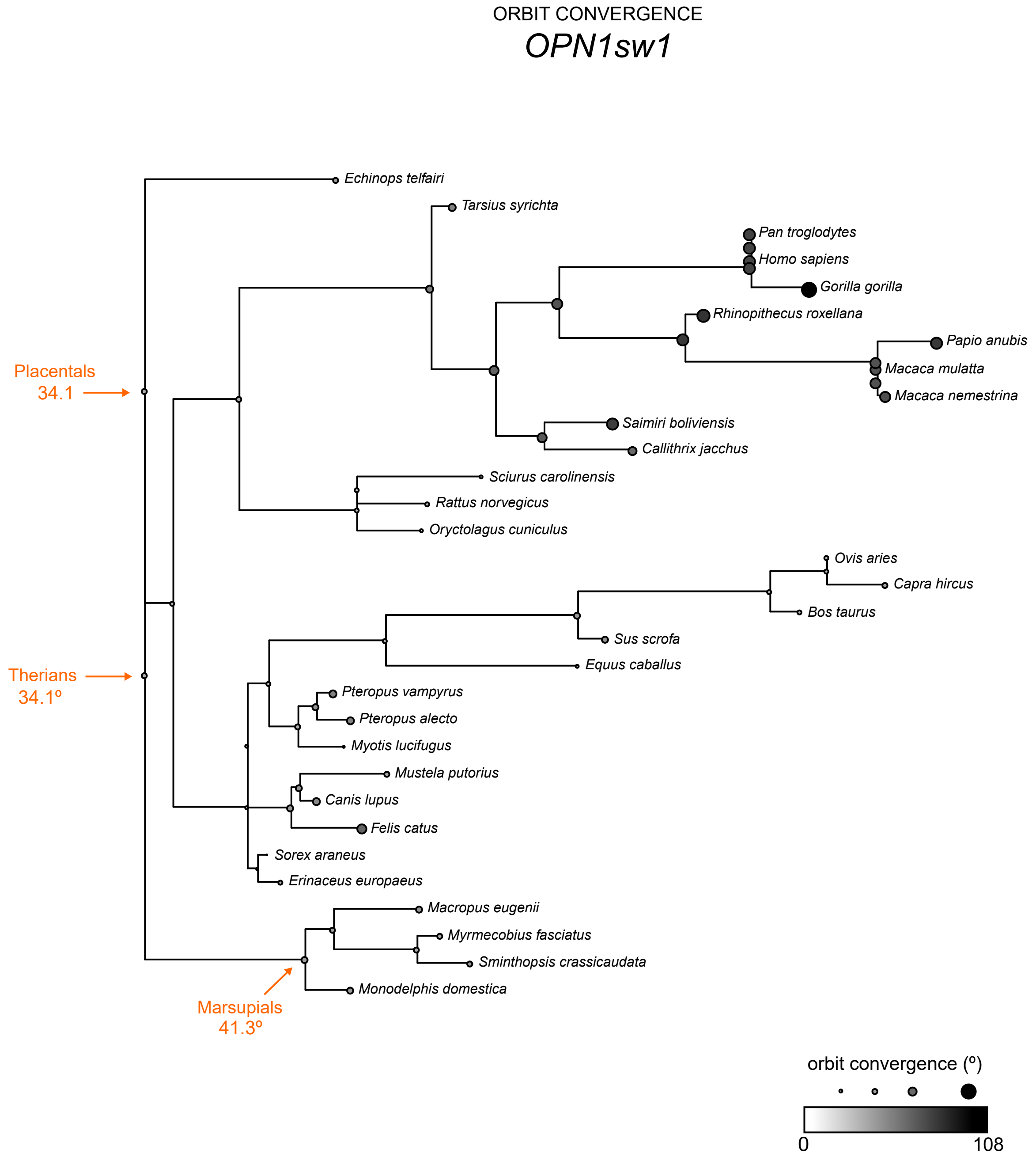

ORBIT CONVERGENCE
OPN1sw1
Echinops telfairi
Tarsius syrichta
Pan troglodytes
Homo sapiens
Gorilla gorilla
Rhinopithecus roxellana
Papio anubis
Macaca mulatta
Macaca nemestrina
Saimiri boliviensis
Callithrix jacchus
Sciurus carolinensis
Rattus norvegicus
Oryctolagus cuniculus
Ovis aries
Capra hircus
Bos taurus
Sus scrofa
Equus caballus
Pteropus vampyrus
Pteropus alecto
Myotis lucifugus
Mustela putorius
Canis lupus
Felis catus
Sorex araneus
Erinaceus europaeus
Macropus eugenii
Myrmecobius fasciatus
Sminthopsis crassicaudata
Monodelphis domestica
Placentals
34.1
Therians
34.1º
Marsupials
41.3º
orbit convergence (º)
0
108

ORBIT CONVERGENCE

OPN1lw

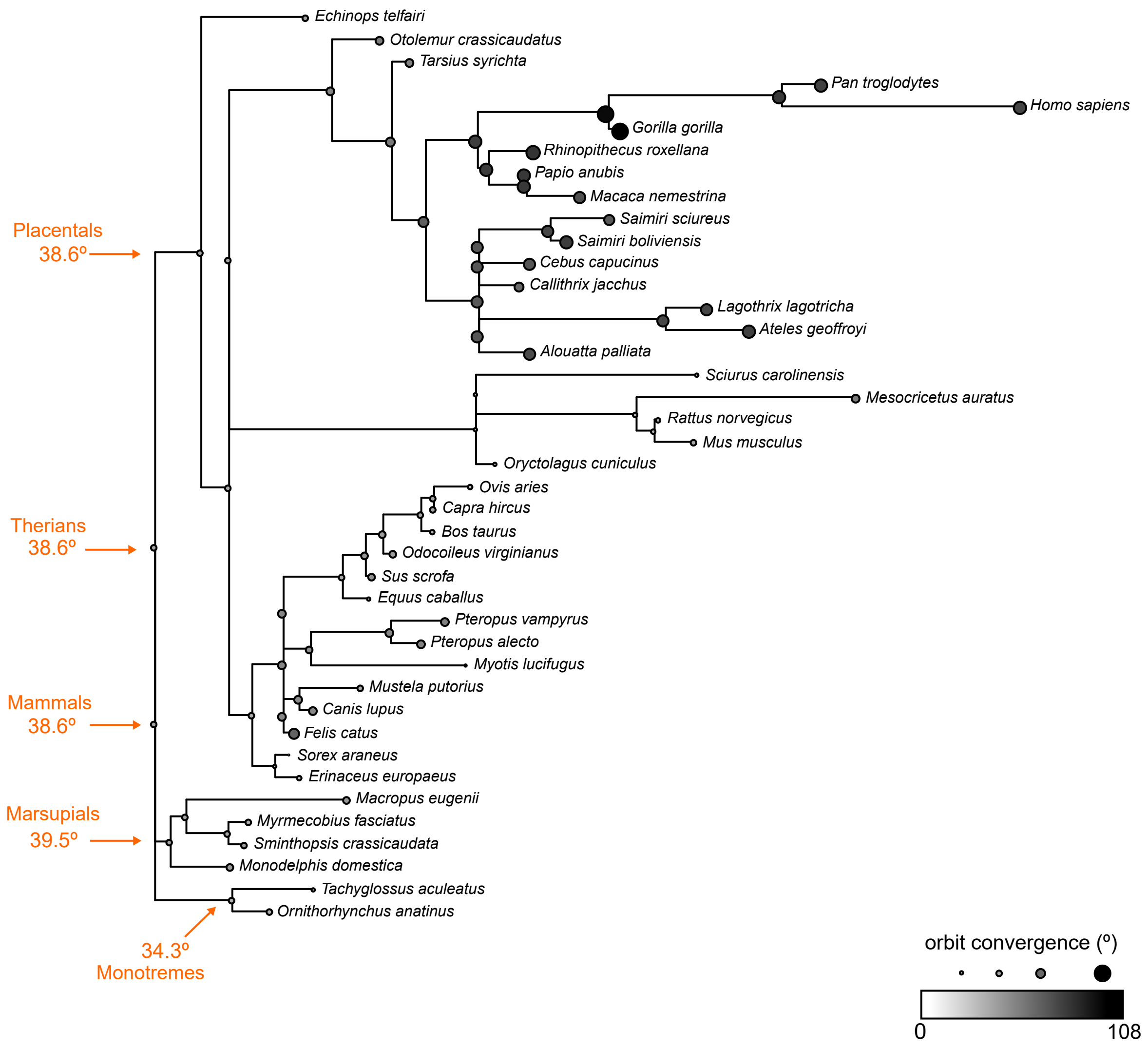

ORBIT CONVERGENCE

OPN3

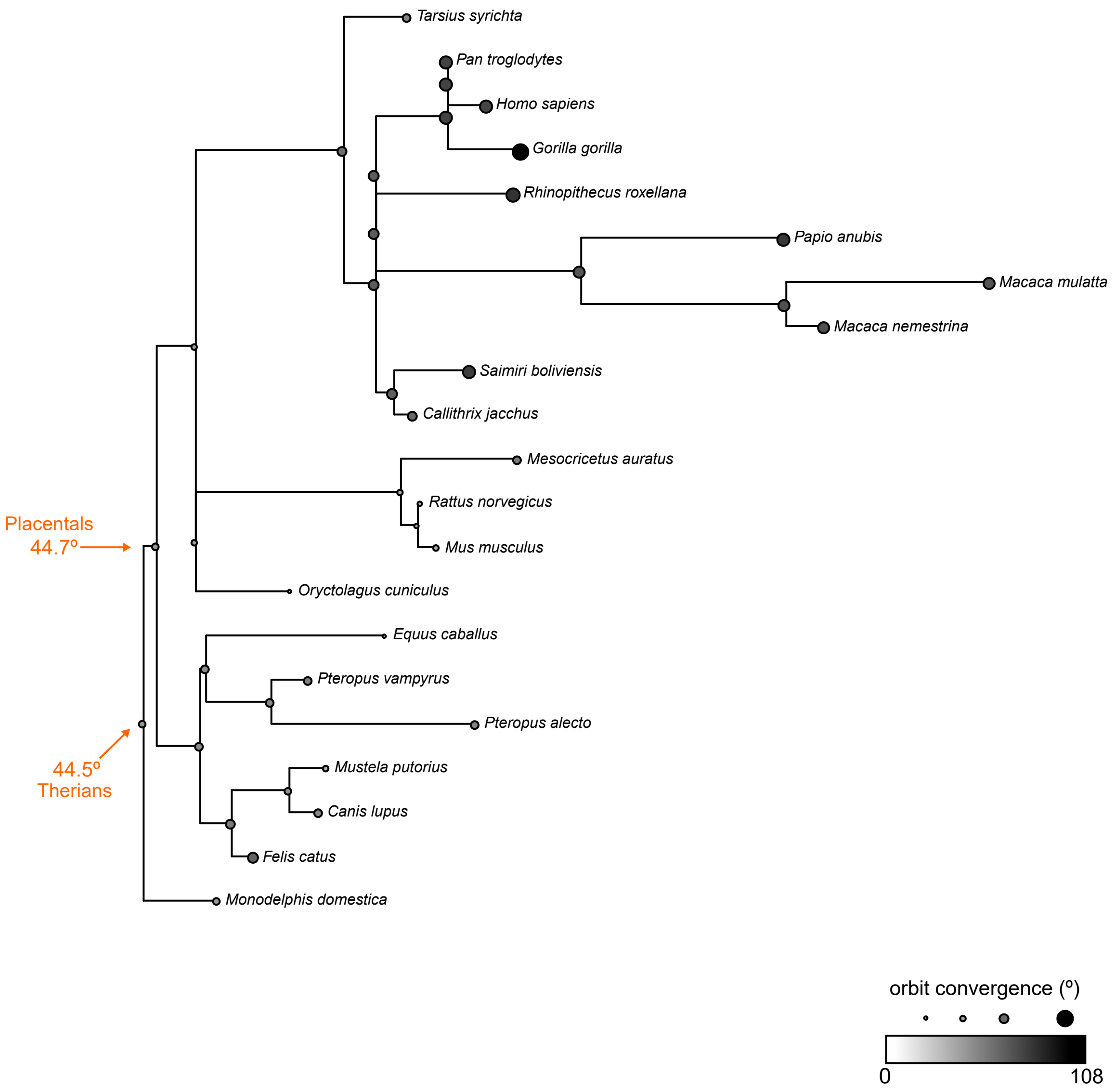

ORBIT CONVERGENCE
OPN5

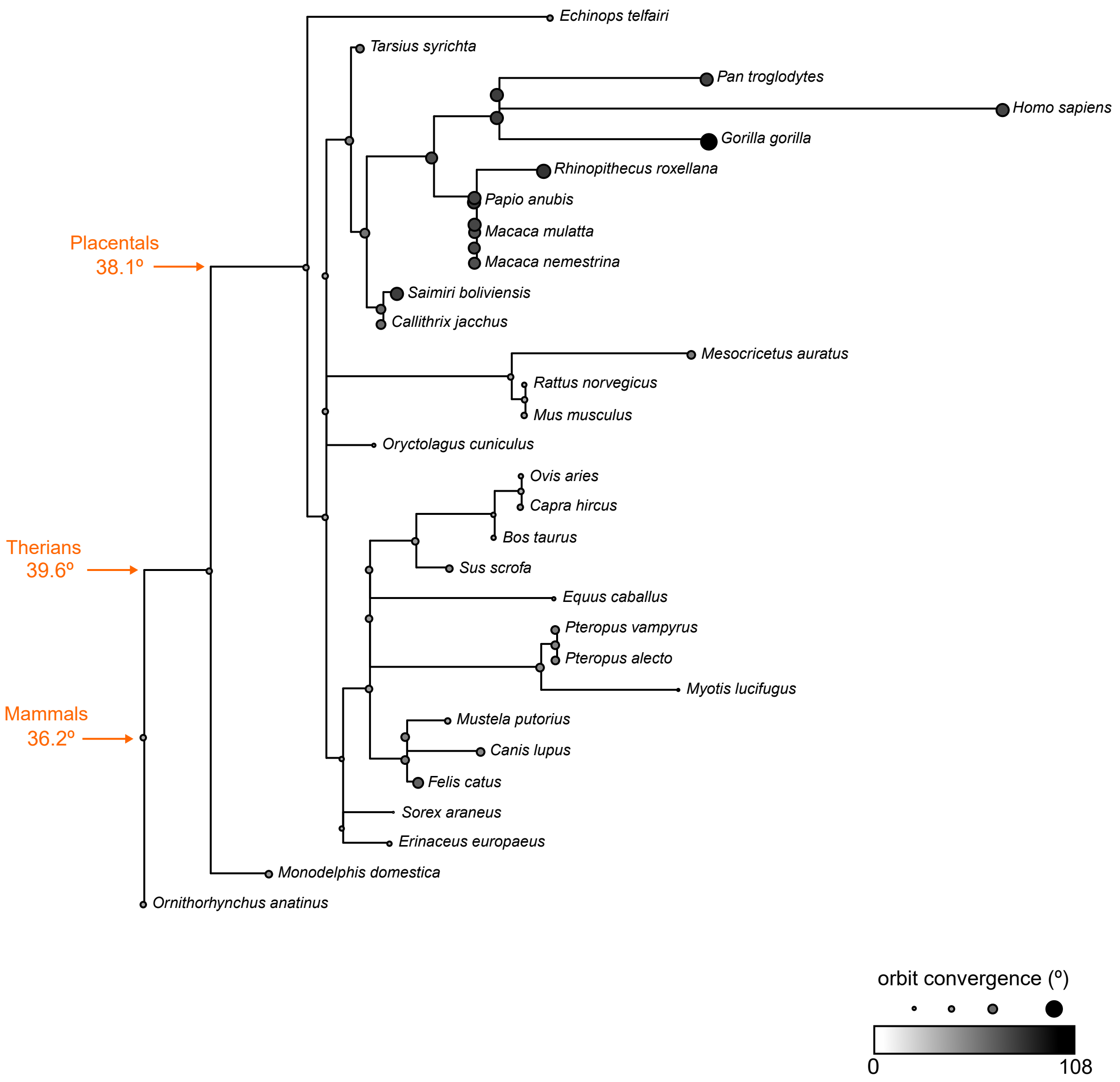

ORBIT CONVERGENCE

RGR

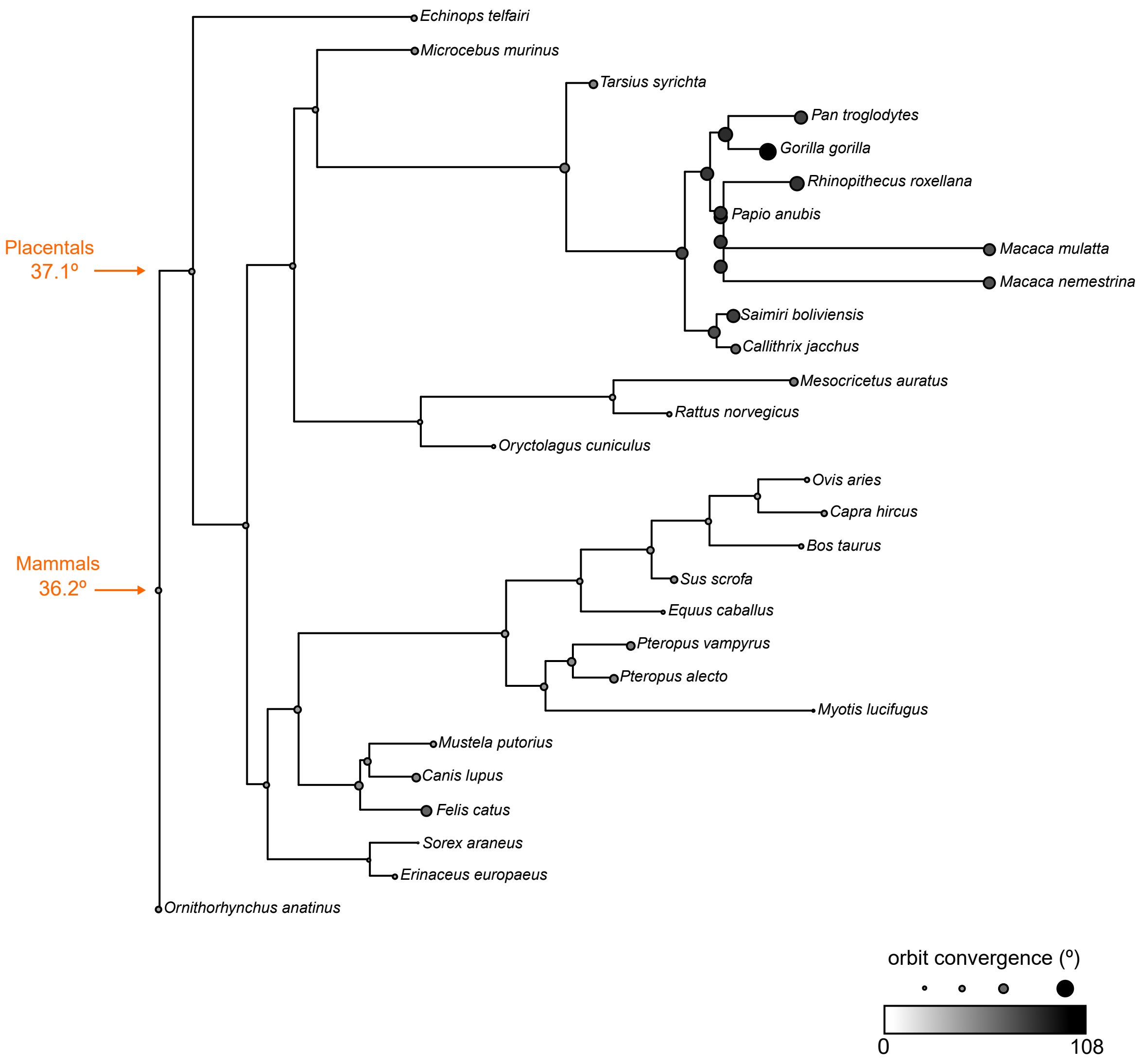

ORBIT CONVERGENCE

RRH

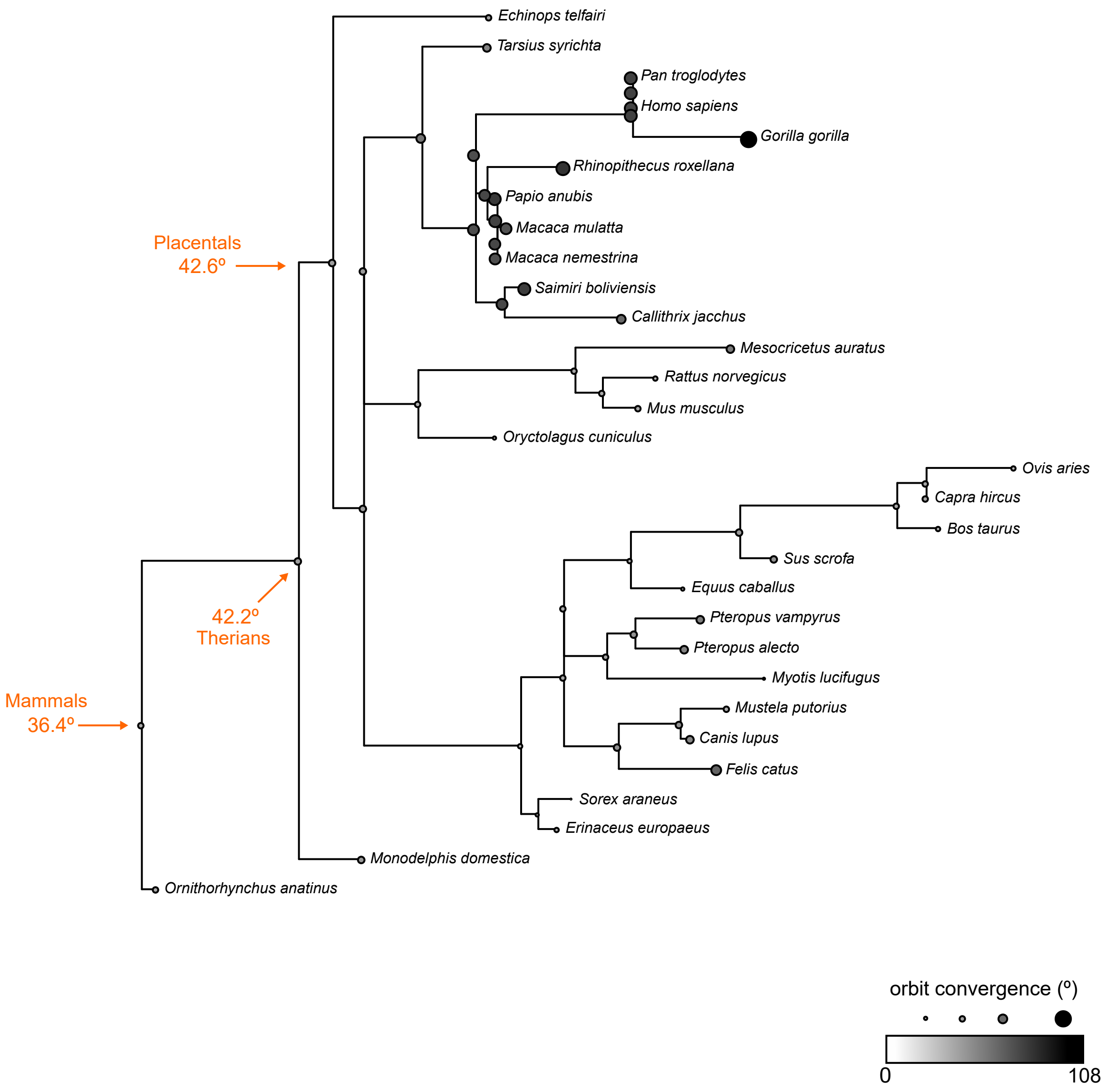

ORBIT CONVERGENCE

OPN4m

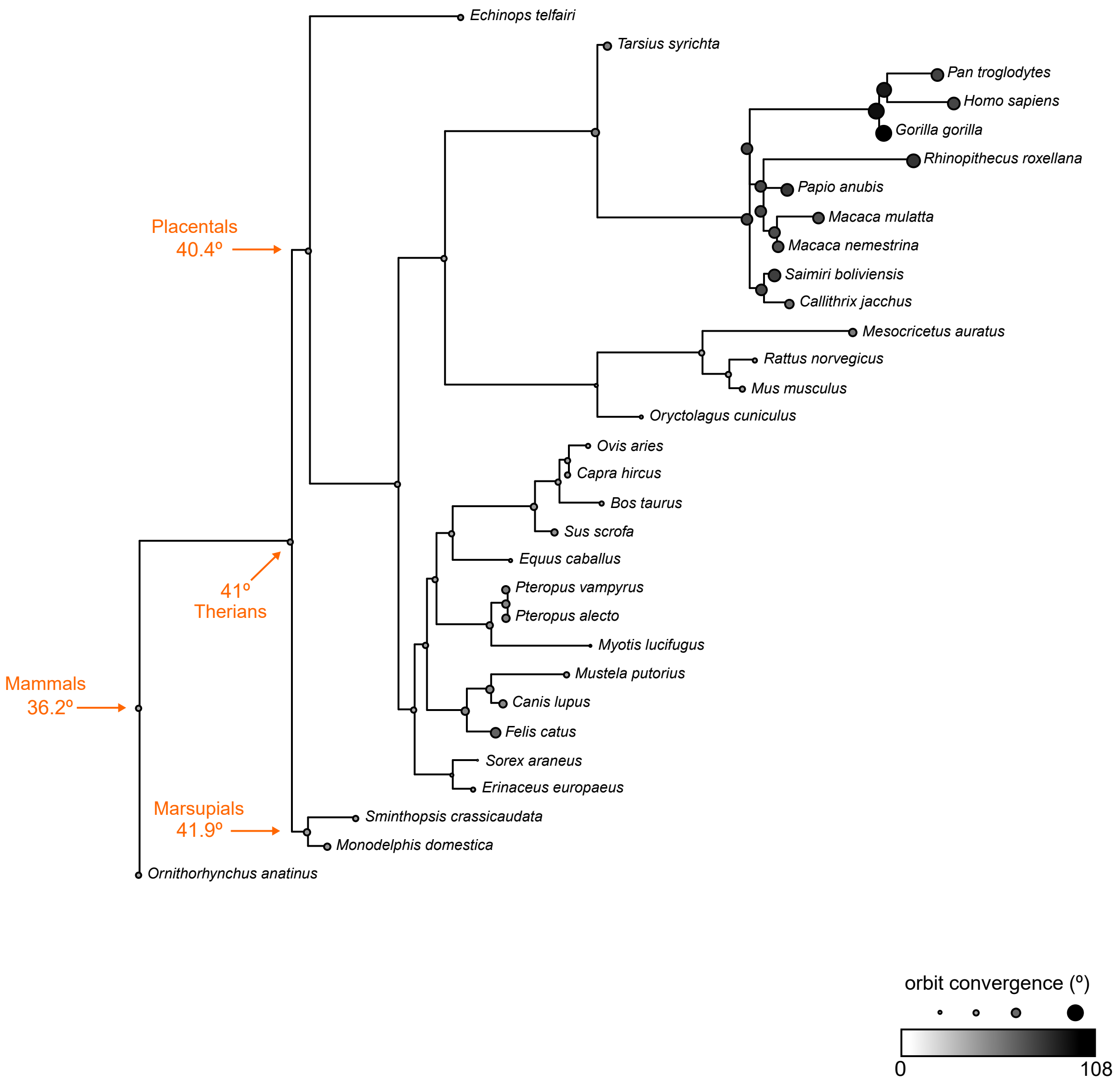

Supplement: Supplementary file 5 — Phylogenetic character mapping for the ancestral reconstructions. Ancestral reconstructions of the activity pattern and sw1-sensitivity are represented in pie charts, each slice representing the probability of each state. Ancestral inferences of the orbit convergence (degrees) and visual acuity (cycles per degree) are represented by circles that change in size and shade of grey according to the character value. The opsin trees were estimated under the assumption of the branch-specific free-ratios model using the Meredith et al. (2011) tree topology. (PDF 1239 kb) [file 12864_2017_4417_MOESM5_ESM.pdf]
