# Supplementary material for: Adaptive genomic evolution of opsins reveals that early mammals flourished in nocturnal environments
Source: BMC Genomics. 2018 Feb 5;19:121. doi: 10.1186/s12864-017-4417-8 (PMC5800076; doi:10.1186/s12864-017-4417-8)
Supplement: Supplementary file 6 — Ancestral character reconstructions of orbit convergence and visual acuity in mammals. The phylogenetic association between each variable and the opsin ω-tree was tested using the Moran’s I hypothesis test (p-value <0.003, Bonferroni corrected for 16 comparisons). Ancestral character reconstructions were performed using the Brownian motion model, excluding the cases in which none (*) or only one (**) species was representing the clades of interest (mammalia, monotremata, eutheria, marsupialia, placentalia). Inferences for each opsin (mean and standard deviation) are shown for each opsin gene tree. The last line of the table summarizes the results considering the mean probability distribution. (PDF 435 kb) [file 12864_2017_4417_MOESM6_ESM.pdf]

Adaptive genomic evolution of opsins reveals that early mammals flourished in nocturnal environments

Rui Borges, Warren E. Johnson, Stephen J. O'Brien, Cidália Gomes, Christopher P. Heesy and Agostinho Antunes

Table S4

Ancestral character reconstructions of orbit convergence and visual acuity in mammals

The phylogenetic association between each variable and the opsin ω-tree was tested using the Moran's I hypothesis test (p-value < 0.003, Bonferroni corrected for 16 comparisons). Ancestral character reconstructions were performed using the Brownian motion model, excluding the cases in which none (*) or only one (**) species was representing the clades of interest (mammals, monotremes, therians, marsupials, placentals). Inferences for each opsin (mean and standard deviation) are shown for each opsin gene tree. The last line of the table summarizes the results considering the mean probability distribution.

A. Visual acuity (cycles per degree, cpd)

	Moran's *I*	Mammals		Monotremes		Therians		Marsupials		Placentals	
RH1	4.66E-04	1.9	5.7	1.9	4.1	5.3	6.5	5.1	7.8	5.5	5.9
OPN1sw1	4.65E-86	*		*		8.4	16.3	2.5	11.5	8.4	16.3
OPN1lw	3.49E-93	4.1	10.3	2.4	10.6	4.1	10.3	3.4	9	7.8	10.6
OPN3	6.74E-14	*		*		5.7	12.8	3.8	9.9	5.7	12.8
OPN5	7.72E-22	1.8	0.4	**		4.3	8.1	4.7	0.4	6.7	8
RGR	2.07E-27	1.8	0.4	**		*		*		4.2	11.1
RRH	7.68E-16	2	11.7	**		6.6	15.8	4.3	11.7	9.7	15.4
OPN4m	3.14E-70	1.8	0.1	**		4.2	5.4	3.1	3.8	5.6	6.2
		2.2	2.8	2.2	5.7	5.5	4.3	3.8	3.3	6.7	4

B. Orbit convergence (degrees, º)

	Moran's *I*	Mammals		Monotremes		Therians		Marsupials		Placentals	
RH1	4.96E-15	35	5.8	34.6	4.2	43.5	6	43.9	6.9	43.4	5.5
OPN1sw1	8.96E-29	*		*		34.1	20.2	41.3	14.3	34.1	20.2
OPN1lw	1.31E-54	38.6	11.2	34.3	10.8	38.6	11.2	39.5	10.5	38.6	11.7
OPN3	1.04E-06	*		*		44.5	15.6	**		44.7	14.7
OPN5	1.42E-13	36.2	0.4	**		39.6	8.8	**		38.1	8.3
RGR	3.87E-18	36.2	0.4	**		*		*		37.1	11.4
RRH	4.31E-12	36.4	11.9	**		42.2	18.2	**		42.6	17.9
OPN4m	5.00E-39	36.2	0.4	**		41	14.8	41.9	11.2	40.4	16.8
		36.4	2.9	34.5	5.8	40.5	5.4	41.7	5.5	39.9	5
