# Supplementary material for: Adaptive genomic evolution of opsins reveals that early mammals flourished in nocturnal environments
Source: BMC Genomics. 2018 Feb 5;19:121. doi: 10.1186/s12864-017-4417-8 (PMC5800076; doi:10.1186/s12864-017-4417-8)

Adaptive genomic evolution of opsins reveals that early mammals flourished in nocturnal environments

Rui Borges, Warren E. Johnson, Stephen J. O'Brien, Cidália Gomes, Christopher P. Heesy and Agostinho Antunes

Figure S3

Orbit convergence vs. activity patterns in mammals

Box plots depicting the association between the orbit convergence (degrees, º) and the activity pattern (nocturnal and diurnal) of extant mammals.

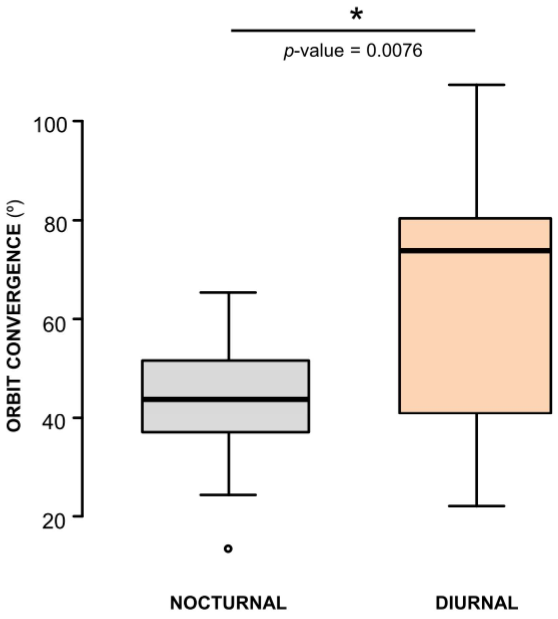

Supplement: Supplementary file 7 — Orbit convergence vs. activity patterns in mammals. Box plots depicting the association between the orbit convergence (degrees, °) and the activity pattern (nocturnal and diurnal) of extant mammals. (PDF 276 kb) [file 12864_2017_4417_MOESM7_ESM.pdf]
