# Supplementary material for: Adaptive genomic evolution of opsins reveals that early mammals flourished in nocturnal environments
Source: BMC Genomics. 2018 Feb 5;19:121. doi: 10.1186/s12864-017-4417-8 (PMC5800076; doi:10.1186/s12864-017-4417-8)
Supplement: Supplementary file 9 — Saturation analysis. The presence of saturation in base substitution for each of the opsin gene alignment was tested by comparing half of the expected theoretical saturation index when assuming full saturation (Iss.c, critical value) with the observed saturation index (Iss). The absence of substitution saturation is verified when Iss is lower than Iss.c for a significant p-value. (PDF 107 kb) [file 12864_2017_4417_MOESM9_ESM.pdf]

Adaptive genomic evolution of opsins reveals that early mammals flourished in nocturnal environments

Rui Borges, Warren E. Johnson, Stephen J. O'Brien, Cidália Gomes, Christopher P. Heesy and Agostinho Antunes

Table S6

Saturation analysis

The presence of saturation in base substitution for each of the opsin gene alignment was tested by comparing half of the expected theoretical saturation index when assuming full saturation (Iss.c, critical value) with the observed saturation index (Iss). Absence of substitution saturation is verified when Iss is lower than Iss.c for a significant p-value.

**Opsin**	**Iss**	**Iss.c**	**t**	***df***	***p*-value**
RH1	0.284	0.751	36.203	1043	0
OPN1sw1	0.307	0.751	34.954	1043	0
OPN1lw	0.34	0.754	27.853	1088	0
OPN4m	0.28	0.77	38.115	1391	0
OPN3	0.392	0.761	24.914	1202	0
RGR	0.236	0.738	33.464	872	0
RRH	0.276	0.749	40.882	1010	0
OPN5	0.189	0.752	52.204	1055	0
